# Large-scale biomedical concept recognition: an evaluation of current automatic annotators and their parameters

**DOI:** 10.1186/1471-2105-15-59

**Published:** 2014-02-26

**Authors:** Christopher Funk, William Baumgartner, Benjamin Garcia, Christophe Roeder, Michael Bada, K Bretonnel Cohen, Lawrence E Hunter, Karin Verspoor

**Affiliations:** 1 Computational Bioscience Program, U. of Colorado School of Medicine, Aurora, CO 80045, USA; 2 Center for Genes, Environment, and Health, National Jewish Health, Denver, CO 80206, USA; 3 Victoria Research Lab, National ICT Australia, Melbourne 3010, Australia; 4 Computing and Information Systems Department, University of Melbourne, Melbourne 3010, Australia

## Abstract

**Background:**

Ontological concepts are useful for many different biomedical tasks. Concepts are difficult to recognize in text due to a disconnect between what is captured in an ontology and how the concepts are expressed in text. There are many recognizers for specific ontologies, but a general approach for concept recognition is an open problem.

**Results:**

Three dictionary-based systems (MetaMap, NCBO Annotator, and ConceptMapper) are evaluated on eight biomedical ontologies in the Colorado Richly Annotated Full-Text (CRAFT) Corpus. Over 1,000 parameter combinations are examined, and best-performing parameters for each system-ontology pair are presented.

**Conclusions:**

Baselines for concept recognition by three systems on eight biomedical ontologies are established (F-measures range from 0.14–0.83). Out of the three systems we tested, ConceptMapper is generally the best-performing system; it produces the highest F-measure of seven out of eight ontologies. Default parameters are not ideal for most systems on most ontologies; by changing parameters F-measure can be increased by up to 0.4. Not only are best performing parameters presented, but suggestions for choosing the best parameters based on ontology characteristics are presented.

## Background

Ontologies have grown to be one of the great enabling technologies of modern bioinformatics, particularly in areas like model organism database curation, where they have facilitated large-scale linking of genomic data across organisms, but also in fields like analysis of high-throughput data [1] and protein function prediction [2,3]. Ontologies have also played an important role in the development of natural language processing systems in the biomedical domain, which can use ontologies both as terminological resources and as resources that provide important semantic constraints on biological entities and events [4]. Ontologies provide such systems with a target conceptual representation that abstracts over variations in the surface realization of terms. This conceptual representation of the content of documents in turn enables development of sophisticated information retrieval tools that organize documents based on categories of information in the documents [5-7].

Finally, ontologies themselves can benefit from concept recognition in text. Yao *et al*. [8] propose new ontology quality metrics that are based on the goodness of fit of an ontology with a domain-relevant corpus. They note that a limitation of their approach is the dependency on tools that establish linkages between ontology concepts and their textual representations.

However, a general approach to recognition of terms from any ontology in text remains a very open research problem. While there exist sophisticated named entity recognition tools that address specific categories of terms, such as genes or gene products [9], protein mutations [10], or diseases [11,12], these tools require targeted training material and cannot generically be applied to recognize arbitrary terms from large, fine-grained vocabularies [6]. Furthermore, as Brewster *et al*. [13] point out, there is often a disconnect between what is captured in an ontology and what can be expected to be explicitly stated in text. This is particularly true for relations among concepts, but it is also the case that concepts themselves can be expressed in text with a huge amount of variability and potentially ambiguity and underspecification [14,15].

The work reported here aims to advance the state of the art in recognizing terms from ontologies with a wide variety of differences in both the structure and content of the ontologies and in the surface characteristics of terms associated with concepts in the ontology. In the course of the work reported in this paper, we evaluate a number of hypotheses related to the general task of finding references to concepts from widely varying ontologies in text. These include the following: 

• Not all concept recognition systems perform equally on natural language texts.

• The best concept recognition system varies from ontology to ontology.

• Parameter settings for a concept recognition system can be optimized to improve performance on a given ontology.

• Linguistic analysis, in particular morphological analysis, affects the performance of concept recognition systems.

To test these hypotheses, we apply a variety of dictionary-based tools for recognizing concepts in text to a corpus in which nearly all of the concepts from a variety of ontologies have been manually annotated. We perform an exhaustive exploration of the parameter spaces for each of these tools and report the performance of thousands of combinations of parameter settings. We experiment with the addition of tools for linguistic analysis, in particular morphological analysis. Along with reporting quantitative results, we give the results of manual error analysis for each combination of concept recognition system and ontology.

The gold standard used is the Colorado Richly Annotated Full-Text (CRAFT) Corpus [16,17]. The full CRAFT corpus consists of 97 completely annotated biomedical journal articles, while the “public release” set, which consists of 67 documents, was used for this evaluation. CRAFT includes over 100,000 concept annotations from eight different biomedical ontologies. Without CRAFT, this large-scale evaluation of concept annotation would not have been possible, due to lack of corpora annotated with a large number of concepts from multiple ontologies.

### Related work

A number of tools and strategies have been proposed for concept annotation in text. These include both tools that are generally applicable to a wide range of terminology resources, and strategies that have been designed specifically for one or a few terminologies. The two most widely used generic tools are the National Library of Medicine’s MetaMap [18] and NBCO’s Open Biomedical Annotator (NCBO Annotator) [19], based on a tool from the University of Michigan called MGREP. Other tools, including Whatizit [20], KnowledgeMap [21,22], CONANN [23], IndexFinder [24,25], Terminizer [26], and Peregrine [27,28] have been created but are not publicly available or appear not to be in widespread use. We therefore focus our analysis in this paper on the NCBO Annotator and MetaMap. In addition, we include ConceptMapper [29,30], a tool that was not specifically developed for biomedical term recognition but rather for flexible look up of terms from a dictionary or controlled vocabulary.

The tools MGREP and MetaMap have been directly compared on several term recognition tasks [19,31]. These studies indicate that MGREP outperforms MetaMap in terms of precision of matching. Both studies also note that MetaMap returns many more annotations than MGREP. Recall is not calculated in either study because the document collections used as input were not fully annotated. By using a completely annotated corpus such as CRAFT, we are able to generate not only precision but recall, which gives a complete picture of the performance of the system.

The Gene Ontology [32] has been the target of several customized methods that take advantage of the specific structure and characteristics of that ontology to facilitate recognition of its constituent terms in text [2,33-36]. In this work, we will not specifically compare these methods to the more generic tools identified above, as they are not applicable to the full range of ontologies that are reflected in the CRAFT annotations.

The CRAFT corpus has been utilized previously in the context of evaluating the recognition of specific categories of terms. Verspoor *et al*. [16] provide a detailed assessment of named entity recognition tool performance for recognition of genes and gene products. As with the work mentionned in the previous paragraph, these are specialized tools with a more targeted approach than we explore in this work, typically requiring substantial amounts of training material tailored to the specific named entity category. We do not repeat those experiments here as they are not relevant to the general problem of recognition of terms from large controlled vocabularies.

### A note on “concepts”

We are aware of the controversies associated with the use of the word “concept” with respect to biomedical ontologies, but the content of the paper is not affected by the conflicting positions on this issue; we use the word to refer to the tuple of namespace, identifier, term(s), definition, synonym(s), and metadata that make up an entry in an ontology.

## Methods

### Corpus

We used version 1.0, released October 19, 2012, of the Colorado Richly Annotated Full Text Corpus (CRAFT) data set [16,17]. The full corpus consists of 97 full-text documents selected from the PubMed Central Open Access subset. Each document in the collection serves as evidence for at least one mouse functional annotation. For this paper we used the “public release” subsection, which consists of 21,000 sentences from 67 articles. There are over 100,000 concept annotations from eight different biomedical ontologies in this public subset. Each annotation specifies the identifier of the concept from the respective ontology along with the beginning and end points of the text span(s) of the annotation.

To fully understand the results presented, it is important to understand how CRAFT was annotated [17]. Here we present three guidelines. First, the text associated with each annotation in CRAFT must be semantically equivalent to the term from the ontology with which it is annotated. In other words, the text, in its context, has the same meaning as the concept used to annotate it. Second, annotations are made to a specific ontology and not to a domain; that is, annotations are created only for concepts explicitly represented in the given ontology and not to concepts that “should” be in the ontology but are not explicitly represented. For example, if the ontology contains a concept representing vesicles, but nothing more specific, a mention of “microvesicles” would not be annotated: Even though it is a type of vesicle, it is not annotated because microvesicles are not explicitly represented in the ontology and annotating this text with the more general vesicle concept would not be semantically equivalent, i.e., information would be lost. Third, only text directly corresponding to a concept is tagged; for example, if the text “mutant vesicles” is seen, “vesicles” is tagged by itself (i.e. without “mutant”) with the vesicle concept. Because only the most specific concept is annotated, there are no subsuming annotations; that is, given an annotation of a text span with a particular concept, no annotations are made within this text span(s) with a more general concept even if they appear in the term. For an example from the Cell Type Ontology, given the text “mesenchymal cell”, this phrase is annotated with “CL:0000134 - mesenchymal cell” but the nested “cell” is not additionally annotated with “CL:0000000 - cell”, as the latter is an ancestor of the former and therefore redundant. There are very specific guidelines as to what text is included in an annotation set out in Bada *et al*. [37].

### Ontologies

The annotations of eight ontologies, representing a wide variety of biomedical terminology, were used for this evaluation: 1–3) The three sub-ontologies of the Gene Ontology (Biological Process, Molecular Function, Cellular Component) [32] 4) the Cell Type Ontology [38] 5) Chemical Entities of Biological Interest Ontology [39] 6) the NCBI Taxonomy [40] 7) the Sequence Ontology [41] and 8) the Protein Ontology [42]. Versions of ontologies used along with descriptive statistics can be seen in Table 1. CRAFT also contains Entrez Gene annotations, but these were analyzed in previous work [16]. The Gene Ontology (GO) aims to standardize the representation of gene and gene product attributes; it consists of three distinct sub-ontologies, which are evaluated separately: Molecular Function, Biological Process, and Cellular Component. The Cell Type Ontology (CL) provides a structured vocabulary for cell types. Chemical Entities of Biological Interest (ChEBI) is focused on molecular entities, molecular parts, atoms, subatomic particles, and biochemical roles and applications. NCBI Taxonomy (NCBITaxon) provides classification and nomenclature of all organisms and types of biological taxa in the public sequence database. The Sequence Ontology (SO) aims to describe the features and attributes of biological sequences. The Protein Ontology (PRO) provides a representation of protein-related entities.

**Table 1:** Characteristics of ontologies evaluated

**Ontology**	**Version**	**# Concepts**	**Avg. term**	**Avg. words**	**Avg. #**	**% Have**	**% Have**	**% Have**
			**length**	**in term**	**synonyms**	**punctuation**	**numerals**	**stop words**
Cell type	25:05:2007	838	20.0 ± 9.5	3.0 ± 1.4	0.5 ± 1.1	11.6	4.8	3.3
Sequence	30:03:2009	1,610	21.6 ± 13.3	3.1 ± 1.0	1.4 ± 1	91.9	6.6	9.3
ChEBI	28:05:2008	19,633	25.5 ± 24.2	4.3 ± 4.8	2.0 ± 2.5	54.8	41.3	0
NCBITaxon	12:07:2011	789,538	24.6 ± 10.2	3.6 ± 2.0	N/A	53.7	56.0	0.3
GO-MF	28:11:2007	7,984	39.1 ± 15.4	4.6 ± 2.2	2.8 ± 4.6	52.8	26.6	2.7
GO-BP	28:11:2007	14,306	40.1 ± 19.0	5.0 ± 2.7	2.1 ± 2.5	23.5	7.0	45.7
GO-CC	28:11:2007	2,047	26.6 ± 14.2	3.6 ± 1.7	0.1 ± 0.9	29.5	14.4	6.8
Protein	22:04:2011	26,807	38.4 ± 18.5	5.5 ± 2.5	3.1 ± 3.2	68.4	74.8	4.3

### Structure of ontology entries

The ontologies used are from the Open Biomedical Ontologies (OBO) [43] flat file format. To help to understand the structure of the file, an entry of a concept from CL is shown below. The only parts of an entry used in our systems are the id, name, and synonym rows. Alternative ways to refer to terms are expressed as synonyms; there are many types of synonyms that can be specified with different levels of relatedness to the concept (exact, broad, narrow, and related). An ontology contain a hierarchy among its terms; these are expressed in the “is_a” entry. Terms described as “ancestors”, “less specific”, or “more general” lie above the specified concept in the hierarchy, while terms described as “more specific” are below the specified concept.

**id:** CL:0000560

**name:** band form neutrophil

**def:** “A late neutrophilic metamyelocyte in which the nucleus is in the form of a curved or coiled band, not having acquired the typical multi lobar shape of the mature neutrophil.”

***synonym:*** “band cell” EXACT

**synonym:** “rod neutrophil” EXACT

**synonym:** “band” NARROW

**is_a:** CL:0000776 ! immature neutrophil

**relationship:** develops_from CL:0000582 neutrophilic metamyelocyte

### A note on obsolete terms

Ontologies are ever changing: new terms are added, modifications are made to others, and others are made obsolete. This poses a problem because obsolete terms are not removed from the ontology, but only marked as obsolete in the obo flat file. The dictionary-based methods used in our analysis do not distinguish between valid or obsolete terms when creating their dictionaries, so obsolete terms may be returned by the systems. A filter was incorporated to remove obsolete terms returned (discussed more below). Not filtering obsolete terms introduces many false positives. For example, the terms “GO:0005574 - DNA” and “GO:0003675 - protein” are both obsolete in the cellular component branch of the Gene Ontology and are mentioned very frequently within the biomedical literature.

### Concept recognition systems

We evaluated three concept recognition systems, NCBO Annotator (NCBO Annotator) [44], MetaMap [18], and ConceptMapper [29,30]. All three systems are publicly available and able to produce annotations for many different ontologies but differ in their underlying implementation and amount of configurable parameters. The full evaluation results are available for download at http://bionlp.sourceforge.net/.

NCBO Annotator is a web service provided by the National Center for Biomedical Ontology (NCBO) that annotates textual data with ontology terms from the UMLS and BioPortal ontologies. The input text is fed into a concept recognition tool (MGREP) and annotations are produced. A wrapper [45] programmatically converts annotations produced by NCBO into xml, which is then imported into our evaluation pipeline. The evaluations from NCBO Annotator were performed in October and November 2012.

MetaMap (MM) is a highly configurable program created to map biomedical text to the UMLS Metathesaurus. MM parses input text into noun phrases and generates variants (alternate spellings, abbreviations, synonyms, inflections and derivations) from these. A candidate set of Metathesaurus terms containing one of the variants is formed, and scores are computed on the strength of mapping from the variants to each candidate term. In contrast to a Web service, MM runs locally; we installed MM v.2011 on a local Linux server. MM natively works with UMLS ontologies, but not all ontologies that we have evaluated are a part of the UMLS. The optional data file builder [46] allows MM to use any ontology as long as they can be formatted as UMLS database tables; therefore, a Perl script was written to convert the ontology obo files to UMLS database tables following the specification in the data file builder overview.

ConceptMapper (CM) is part of the Apache UIMA [47] Sandbox and is available at http://uima.apache.org/d/uima-addons-current/ConceptMapper. Version 2.3.1 was used for these experiments. CM is a highly configurable dictionary lookup tool implemented as a UIMA component. Ontologies are mapped to the appropriate dictionary format required by ConceptMapper. The input text is processed as tokens; all tokens within a span (sentence) are looked up in the dictionary using a configurable lookup algorithm.

### Parameter exploration

Each system’s parameters were examined and configurable parameters were chosen. Table 2 gives a list of each system with the chosen parameters along with a brief description and possible values. The list of stop words used is provided in Additional file 1.

**Table 2:** System parameter description and values

**NCBO Annotator parameters**	
**Parameter**	**Description and possible values**
wholeWordOnly	Term recognition must match whole words - (YES, NO)
filterNumber	Specifies whether the entity recognition step should filter numbers - (YES, NO)
stopWords	List of stop words to exclude from matching - (PubMed - commonly found terms from PubMed (included as Additional file 1), NONE)
stopWordsCaseSensitive	Whether stop words are case sensitive - (YES, NO)
minTermSize	Specifies minimum length of terms to be returned - (ONE, THREE, FIVE)
withSynonyms	Whether to include synonyms in matching - (YES, NO)
**MetaMap parameters**	
**Parameter**	**Description and possible values**
model	Determines which data model is used - (STRICT - lexical, manual, and syntactic filtering are applied, RELAXED - lexical and manual filtering are used)
gaps	Specifies how to handle gaps in terms when matching - (ALLOW, NONE)
wordOrder	Specifies how to handle word order when matching - (ORDER MATTERS, IGNORE)
acronymAbb	Determines which generated acronym or abbreviations are used - (NONE, DEFAULT, UNIQUE - restricts variants to only those with unique expansions)
derivationalVars	Specifies which type of derivational variants will be used - (NONE, ALL, ONLY ADJ NOUN)
scoreFilter	MetaMap reports a score from 0–1000 for every match, with 1000 being the highest, those matches with scores ≤ will be returned - (0, 600, 800, 1000)
minTermSize	Specifies minimum length of terms to be returned - (ONE, THREE, FIVE)
**ConceptMapper parameters**	
**Parameter**	**Description and possible values**
searchStrategy	Specifies the dictionary lookup strategy - (CONTIGUOUS - longest match of contiguous tokens, SKIP ANY - returns longest match of not-necessarily contiguous tokens and next lookup begin in next span, SKIP ANY ALLOW OVERLAP - returns longest match of not-necessarily contiguous tokens in the span and next lookup begin after next token)
caseMatch	Specifies the case folding mode to use - (IGNORE - fold everything to lower case, INSENSITIVE - fold only tokens with initial caps to lowercase, SENSITIVE - no folding, FOLD DIGIT - fold only tokens with digits to lower case)
stemmer	Name of the stemmer to use before matching - (Porter - classic stemmer that removes common morphological and inflectional endings from Engish words, BioLemmatizer - domain specific lemmatization tool for the morphological analysis of biomedical literature presented in Liu *et al*. [48], NONE)
orderIndependentLookup	Specifies if ordering of tokens within a span can be ignored - (TRUE, FALSE)
findAllMatches	Specifies if all matches will be returned - (TRUE, FALSE - only the longest match will be returned)
stopWords	List of stop words to exclude from matching - (PubMed - commonly found terms from PubMed (included as Additional file 1), NONE)
synonyms	Specifies which synonyms will be included when creating the dictionary - (EXACT ONLY, ALL)

Parameters that were evaluated for each system along with a description and possible values are listed in all capital letters. For the most part, parameters are self-explanatory, but for more information see documentation for each system. CM [29], NCBO Annotator [44], MM [18].

### Evaluation pipeline

An evaluation pipeline for each system was constructed and run in UIMA [49]. MM produces annotations separate from the evaluation pipeline; UIMA components were created to load the annotations before evaluation. NCBO Annotator is able to produce annotations and evaluate them within the same pipeline, but NCBO Annotator annotations were cached to avoid hitting the Web service continually. Like MM, a separate analysis engine was created to load annotations before evaluation. CM produces annotations and evaluates them in a single pipeline.

Evaluation pipelines for each system have a similar structure. First, the gold standard is loaded; then, the system’s annotations are loaded, obsolete annotations are removed, and finally a comparison is made. CRAFT was not annotated with obsolete terms, so the obsolete terms filtered out are those that are obsolete in the version of the ontology used to annotate CRAFT.

CM and MM dictionaries were created with the versions of the ontologies that were used to annotate CRAFT. Since NCBO Annotator is a Web service, we do not have control over the versions of ontologies used; it uses newer versions with more terms. To remove spurious terms not present in the ontologies used to annotate CRAFT, a filter was added to the NCBO Annotator evaluation pipeline. The NCBO Annotator specific filter removes terms not present in the version used to annotate CRAFT and ensures that the term is not obsolete in the version used to annotate CRAFT. Because the versions of the ontologies used in CRAFT are older, it may be the case that some terms annotated in CRAFT are obsolete in the current versions. All systems were restricted to only using valid terms from the versions of the ontology used to annotate CRAFT.

All comparisons were performed using a STRICT comparator, which means that ontology ID and span(s) of a given annotation must match the gold-standard annotation exactly to be counted correct. A STRICT comparator was chosen because it was our desire to see how well automated methods can recreate exact human annotations. A pitfall of the using a STRICT comparator is that a distinction cannot be made between erroneous terms vs. those along the same hierarchical lineage; both are counted as fully incorrect in our analysis. For example, if the gold standard annotation is “GO:0005515 - protein binding” and “GO:0005488 - binding” is returned by a system, partial credit should be given because “binding” is an ancestor of “protein binding”. Future comparisons could address this limitation by accounting for the hierarchical relationship in the ontology by counting those less specific terms as partially correct by using hierarchical precision/recall/F-measure as seen in Verspoor *et al*. [50].

The output is a text file for each parameter combination listing true positives (TP), false positives (FP), and false negatives (FN) for each document as well as precision (P), recall (R), and F-measure (F) (Calculations of P, R, and F can be seen in formulas 1, 2, and 3). Precision, recall, and F-measure are calculated over all annotations across all documents in CRAFT, i.e. as a *micro-average*.

(1) $$\begin{array}{ll} P & =\frac{\text{TP}}{\text{TP}+\text{FP}}\; \end{array}$$

(2) $$\begin{array}{ll} R & =\frac{\text{TP}}{\text{TP}+\text{FN}}\; \end{array}$$

(3) $$\begin{array}{ll} F & =2*\frac{P*R}{P+R}\; \end{array}$$

### Statistical analysis

The Kruskal-Wallis statistical method was chosen to test significance for all our comparisons because it is a non-parametric test that identifies differences between ranked group of variables. It is appropriate for our experiments because we do not assume our data follows any particular distribution and desire to determine if the distribution of scores from a particular experimental condition, such as tool or parameters, are different from the others. The implementation built into R was used (*kruskal.test*). Kruskal-Wallis was applied in three different ways: 

1. For each ontology, Kruskal-Wallis was used to determine if there is a significant difference in F-measure performance between tools. The mean and variance was computed across all parameter combinations for a given tool, calculated at the corpus level using the micro-average F-measure and provided as input to Kruskal-Wallis.

2. For each tool, Kruskal-Wallis was used to determine if there is a difference in performance between parameter values for each parameter. The mean and variance was computed across all parameter values for a given parameter, calculated at the corpus level using the micro-average F-measure.

3. Results from Kruskal-Wallis only determine if there is a difference between the groups but does not provide insight into how many differences or between which groups a difference exists. When a significant difference was seen between three or more groups, Kruskal-Wallis was used between a *post hoc* test to identify the significantly different group(s).

Significance is determined at a 99% level, *α*=0.01; because there are multiple comparisons, a Bonferroni correction was used, and the new significance level is *α*=0.00036.

### Analysis of results files

For each ontology-system pair, an analysis was performed on the maximum F-measure parameter combination. We did not analyze every annotation produced by all systems but made sure to account for ∼70–80% of them. By performing the analysis this way, we are concentrating on the general errors and terms missed rather than rare errors.

For each maximum F-measure parameter combination file, the top 50–150 (grouped by ontology ID and ranked by number of annotations for each ID) of each true positive (TP), false positive (FP), and false negative (FN) were analyzed by separating them into groups of like annotations. For example, the types of bins that FPs fall into are: “errors from variants”, “errors from ambiguous synonyms”, “errors due to identifying less specific concepts”, etc., and are different than the bins into which TPs or FNs are categorized.

Because we evaluated all parameter combinations, we were able to examine the impact of single parameters by holding all other parameters constant. The maximum F-measure producing parameter combination result file and the complementary result file with varied parameter were run through a graphical difference program, DiffMerge, to examine the annotations found/lost by varying the parameter. Examples mentioned in the Results and discussion are from this comparison.

## Results and discussion

Results and discussion are broken down by ontology and then by tool. For each ontology we present three different levels of analysis: 

1. At the ontology level. This provides a synopsis of overall performance for each system with comments about common terms correct (TPs), errors (FPs), and categories missed (FNs). Specific examples are taken from the top-performing, highest F-measure parameter combination.

2. A high-level parameter analysis, performed over all parameter combinations. This allows for observation about impact on performance seen by manipulating parameter values, presented as ranges of impact. (Presented in Additional file 2).

3. A low-level analysis obtained from examining individual result files gives insight into specific terms or categories of terms that are affected by manipulating parameters. (Presented in Additional file 2).

Within a particular ontology, each system’s performance is described. The most impactful parameters are explored further and examples from variations on maximum F-measure combination are provided to show the effect they have on matching. Results presented as numbers of annotations are of this type of analysis. We end the Results and discussion Section with overall parameter analysis and suggestions for parameters on any ontology.

**Figure 1:**
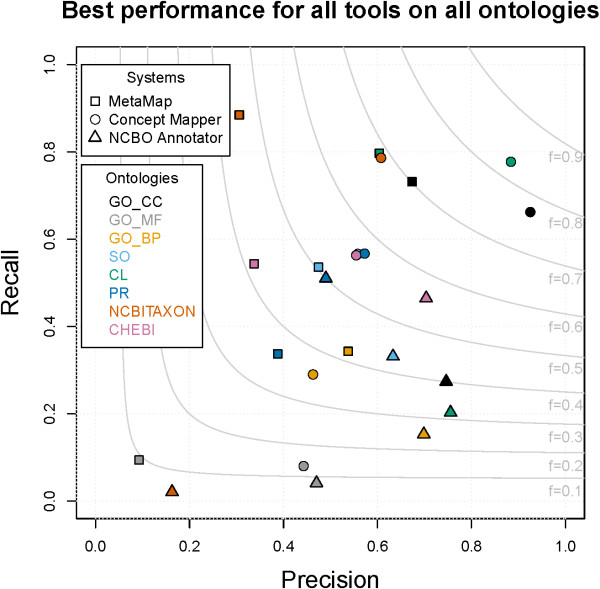
**Maximum F-measure for each system-ontology pair.** A wide range of maximum scores is seen for each system within each ontology.

The best-performing result for each system-ontology pair is presented in Figure 1. There is a wide range of F-measures for all ontologies, from < 0.10 to 0.83. Not only is there a wide range when looking at all ontologies, but a wide range can be seen within each ontology. Two of our hypotheses are supported by this analysis: we can see that not all concept recognition systems perform equally, and the best concept recognition system varies from ontology to ontology.

### Best parameters

Based on analysis, the suggested parameters for maximum performance for each ontology-system pair can be seen in Tables 3 and 4.

**Table 3:** Best performing parameter combinations for CL and GO subsections

**Cell Type Ontology (CL)**					
**NCBO Annotator**		**MetaMap**		**ConceptMapper**	
**Parameter**	**Value**	**Parameter**	**Value**	**Parameter**	**Value**
wholeWordOnly	YES	model	ANY	searchStrategy	CONTIGUOUS
filterNumber	ANY	gaps	NONE	caseMatch	INSENSITIVE
stopWords	ANY	wordOrder	ORDER MATTERS	stemmer	Porter/BioLemmatizer
SWCaseSensitive	ANY	acronymAbb	DEFAULT/UNIQUE	stopWords	NONE
minTermSize	ONE/THREE	derivationalVariants	ALL	orderIndLookup	OFF
withSynonyms	YES	scoreFilter	0	findAllMatches	NO
		minTermSize	1/3	synonyms	EXACT ONLY
**Gene Ontology - Cellular Component (GO_CC)**					
**NCBO Annotator**		**MetaMap**		**ConceptMapper**	
**Parameter**	**Value**	**Parameter**	**Value**	**Parameter**	**Value**
wholeWordOnly	YES	model	ANY	searchStrategy	CONTIGUOUS
filterNumber	ANY	gaps	NONE	caseMatch	INSENSITIVE
stopWords	ANY	wordOrder	ORDER MATTERS	stemmer	Porter
SWCaseSensitive	ANY	acronymAbb	DEFAULT/UNIQUE	stopWords	NONE
minTermSize	ONE/THREE	derivationalVariants	ANY	orderIndLookup	OFF
withSynonyms	ANY	scoreFilter	0/600	findAllMatches	NO
		minTermSize	1/3	synonyms	EXACT ONLY
**Gene Ontology - Molecular Function (GO_MF)**					
**NCBO Annotator**		**MetaMap**		**ConceptMapper**	
**Parameter**	**Value**	**Parameter**	**Value**	**Parameter**	**Value**
wholeWordOnly	NO	model	ANY	searchStrategy	CONTIGUOUS
filterNumber	ANY	gaps	NONE	caseMatch	ANY
stopWords	ANY	wordOrder	ORDER MATTERS	stemmer	BioLemmatizer
SWCaseSensitive	ANY	acronymAbb	DEFAULT/UNIQUE	stopWords	NONE
minTermSize	ANY	derivationalVariants	ANY	orderIndLookup	OFF
withSynonyms	NO	scoreFilter	0/600	findAllMatches	NO
		minTermSize	1/3	synonyms	EXACT ONLY
**Gene Ontology - Biological Process (GO_BP)**					
**NCBO Annotator**		**MetaMap**		**ConceptMapper**	
**Parameter**	**Value**	**Parameter**	**Value**	**Parameter**	**Value**
wholeWordOnly	YES	model	ANY	searchStrategy	CONTIGUOUS
filterNumber	ANY	gaps	NONE	caseMatch	INSENSITIVE
stopWords	ANY	wordOrder	ORDER MATTERS	stemmer	Porter
SWCaseSensitive	ANY	acronymAbb	ANY	stopWords	NONE
minTermSize	ANY	derivationalVariants	ADJ NOUN VARS	orderIndLookup	OFF
withSynonyms	YES	scoreFilter	0	findAllMatches	NO
		minTermSize	5	synonyms	ALL

Suggested parameters to use that correspond to best score on CRAFT. Parameters where choices don’t seem to make a difference in performance are represented as “ANY”.

**Table 4:** Best performing parameter combinations for SO, ChEBI, NCBITaxon, and PRO

**Sequence Ontology (SO)**					
**NCBO Annotator**		**MetaMap**		**ConceptMapper**	
**Parameter**	**Value**	**Parameter**	**Value**	**Parameter**	**Value**
wholeWordOnly	YES	model	STRICT	searchStrategy	CONTIGUOUS
filterNumber	ANY	gaps	NONE	caseMatch	INSENSITIVE
stopWords	ANY	wordOrder	ANY	stemmer	Porter/BioLemmatizer
SWCaseSensitive	ANY	acronymAbb	DEFAULT/UNIQUE	stopWords	NONE
minTermSize	THREE	derivationalVariants	NONE	orderIndLookup	OFF
withSynonyms	YES	scoreFilter	600	findAllMatches	NO
		minTermSize	3	synonyms	EXACT ONLY
**Protein Ontology (PRO)**					
**NCBO Annotator**		**MetaMap**		**ConceptMapper**	
**Parameter**	**Value**	**Parameter**	**Value**	**Parameter**	**Value**
wholeWordOnly	YES	model	ANY	searchStrategy	ANY
filterNumber	ANY	gaps	NONE	caseMatch	CASE FOLD DIGITS
stopWords	PubMed	wordOrder	ANY	stemmer	NONE
SWCaseSensitive	ANY	acronymAbb	DEFAULT/UNIQUE	stopWords	NONE
minTermSize	ONE/THREE	derivationalVariants	NONE	orderIndLookup	OFF
withSynonyms	YES	scoreFilter	600	findAllMatches	NO
		minTermSize	3/5	synonyms	ALL
**NCBI Taxonomy**					
**NCBO Annotator**		**MetaMap**		**ConceptMapper**	
**Parameter**	**Value**	**Parameter**	**Value**	**Parameter**	**Value**
wholeWordOnly	YES	model	ANY	searchStrategy	SKIP ANY/ALLOW
filterNumber	ANY	gaps	NONE	caseMatch	ANY
stopWords	ANY	wordOrder	ORDER MATTERS	stemmer	BioLemmatizer
SWCaseSensitive	ANY	acronymAbb	DEFAULT/UNIQUE	stopWords	PubMed
minTermSize	FIVE	derivationalVariants	NONE	orderIndLookup	OFF
withSynonyms	ANY	scoreFilter	0/600	findAllMatches	NO
		minTermSize	3	synonyms	EXACT ONLY
**ChEBI**					
**NCBO Annotator**		**MetaMap**		**ConceptMapper**	
**Parameter**	**Value**	**Parameter**	**Value**	**Parameter**	**Value**
wholeWordOnly	YES	model	STRICT	searchStrategy	CONTIGUOUS
filterNumber	ANY	gaps	NONE	caseMatch	ANY
stopWords	ANY	wordOrder	ORDER MATTERS	stemmer	BioLemmatizer
SWCaseSensitive	ANY	acronymAbb	DEFAULT/UNIQUE	stopWords	NONE
minTermSize	ONE/THREE	derivationalVariants	NONE	orderIndLookup	OFF
withSynonyms	YES	scoreFilter	0/600	findAllMatches	YES
		minTermSize	5	synonyms	EXACT ONLY

Suggested parameters to use that correspond to best score on CRAFT. Parameters where choices don’t seem to make a difference in performance are represented as “ANY”.

### Cell Type Ontology

**Figure 2:**
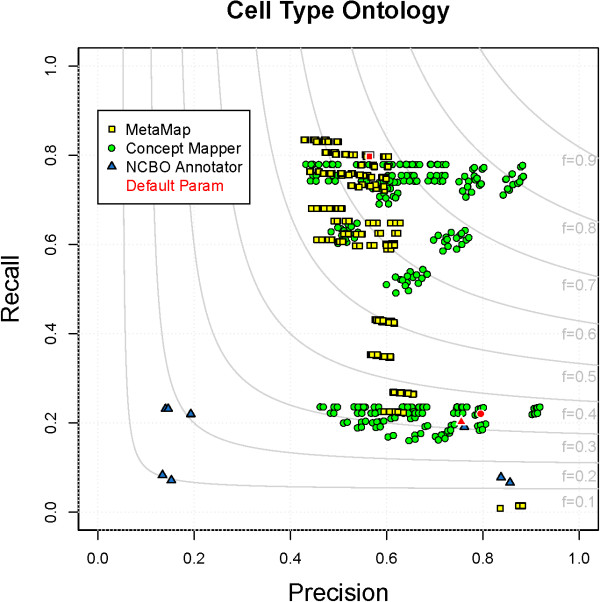
**All parameter combinations for CL.** The distribution of all parameter combinations for each system on CL. (MetaMap - yellow square, ConceptMapper - green circle, NCBO Annotator - blue triangle, default parameters - red).

The Cell Type Ontology (CL) was designed to provide a controlled vocabulary for cell types from many different prokaryotic, fungal, and eukaryotic organisms. Out of all ontologies annotated in CRAFT, it is the smallest, terms are the simplest, and there are very few synonyms (Table 1). The highest F-measure seen on any ontology is on CL. CM is the top performer (F = 0.83), MM performs second best (F = 0.69), and NCBO Annotator is the worst performer (F = 0.32). Statistics for the best scores can be seen in Table 5. All parameter combinations for each system on CL can be seen in Figure 2.

**Table 5:** Best performance for each ontology-system pair

**Cell Type Ontology (CL)**						
**System**	**F-measure**	**Precision**	**Recall**	**# TP**	**# FP**	**# FN**
NCBO Annotator	0.32	0.76	0.20	1169	379	4591
MetaMap	0.69	0.61	0.80	4590	3010	1170
**ConceptMapper**	0.83	0.88	0.78	4478	592	1282
**Gene Ontology - Cellular Component (GO_CC)**						
**System**	**F-measure**	**Precision**	**Recall**	**# TP**	**# FP**	**# FN**
NCBO Annotator	0.40	0.75	0.27	2287	779	6067
MetaMap	0.70	0.67	0.73	6111	2969	2341
**ConceptMapper**	0.77	0.92	0.66	5532	452	2822
**Gene Ontology - Molecular Function (GO_MF)**						
**System**	**F-measure**	**Precision**	**Recall**	**# TP**	**# FP**	**# FN**
NCBO Annotator	0.08	0.47	0.04	173	195	4007
MetaMap	0.09	0.09	0.09	393	3846	3787
**ConceptMapper**	0.14	0.44	0.08	337	425	3834
**Gene Ontology - Biological Process (GO_BP)**						
**System**	**F-measure**	**Precision**	**Recall**	**# TP**	**# FP**	**# FN**
NCBO Annotator	0.25	0.70	0.15	2592	1120	14321
**MetaMap**	0.42	0.53	0.34	5802	4994	11111
ConceptMapper	0.36	0.46	0.29	4909	5710	12004
**Sequence Ontology (SO)**						
**System**	**F-measure**	**Precision**	**Recall**	**# TP**	**# FP**	**# FN**
NCBO Annotator	0.44	0.63	0.33	7056	4094	14231
MetaMap	0.50	0.47	0.54	11402	12634	9885
**ConceptMapper**	0.56	0.56	0.57	12059	9560	9228
**ChEBI**						
**System**	**F-measure**	**Precision**	**Recall**	**# TP**	**# FP**	**# FN**
**NCBO Annotator**	0.56	0.7	0.46	3782	1595	4355
MetaMap	0.42	0.36	0.50	4424	8689	3717
**ConceptMapper**	0.56	0.55	0.56	4583	3687	3554
**NCBI Taxonomy**						
**System**	**F-measure**	**Precision**	**Recall**	**# TP**	**# FP**	**# FN**
NCBO Annotator	0.04	0.16	0.02	157	807	7292
MetaMap	0.45	0.31	0.88	6587	14954	862
**ConceptMapper**	0.69	0.61	0.79	5857	3793	1592
**Protein Ontology (PRO)**						
**System**	**F-measure**	**Precision**	**Recall**	**# TP**	**# FP**	**# FN**
NCBO Annotator	0.50	0.49	0.51	7958	8288	7636
MetaMap	0.36	0.39	0.34	5255	8307	10339
**ConceptMapper**	0.57	0.57	0.57	8843	6620	6751

Maximum F-measure for each system on each ontology. Bolded systems produced the highest F-measure.

Annotations from CL in CRAFT are heavily weighted towards the root node, “CL:0000000 - cell”; it is annotated over 2,500 times and makes up ∼44% of all annotations. To test whether annotations of “cell” introduced a bias, all annotations of CL:0000000 were removed and re-evaluated. (Results not shown here.) We see a decrease in F-measure of 0.08 for all systems and are able to identify similar trends in the effect of parameters when “cell” is not included. We can conclude that “cell” annotations do not introduce any bias.

Precision on CL is good overall, the highest being CM (0.88) and the lowest being MM (0.60), with NCBO Annotator in the middle (0.76). Most of the FPs found are due to partial term matching. “CL:0000000 - cell” makes up more than 50% of total FPs because it is contained in many terms and is mistakenly annotated when a more specific term cannot be found. Besides “cell”, terms recognized that are less specific than the gold standard are “CL:0000066 - epithelial cell” instead of “CL:0000082 - lung epithelial cell” and “CL:0000081 - blood cell” instead of “CL:0000232 - red blood cell”. MM finds more FPs than the other systems, many of these due to abbreviations. For example, MM incorrectly annotates the span “ES cells” with “CL:0000352 - epiblast cell” and “CL:0000034: stem cell”. By utilizing abbreviations, MM correctly annotates “NCC” with “CL:0000333 - neural crest cell”, which the other two systems do not find.

Recall for CM and MM are over 0.8 while NCBO Annotator is 0.2. The low recall seen from NCBO Annotator is due to the fact that it is unable to recognize plurals of terms unless they are explicitly stated in the ontology; it correctly finds “melanocyte” but does not recognize “melanocytes”, for example. Because CL is small and its terms are quite simple, there are only two main categories of terms missed: missing synonyms and conjunctions. The biggest category is insufficient synonyms. We find “cone” and “cone photoreceptor” annotated with “CL:0000573 - retinal cone cell” and “photoreceptor(s)” annotated with “CL:0000210 - photoreceptor cell”; these two examples make up 33% (400 out of 1,200) of annotations missed by all systems. No systems found any annotations that contained conjunctions. For example, for the text span “retinal bipolar, ganglion, and rod cells”, three cell types are annotated in CRAFT: “CL:0000748 - retinal bipolar neuron”, “CL:0000740 - retinal ganglion cell”, and “CL:0000604 - retinal rod cell”.

### Gene Ontology - Cellular Component

**Figure 3:**
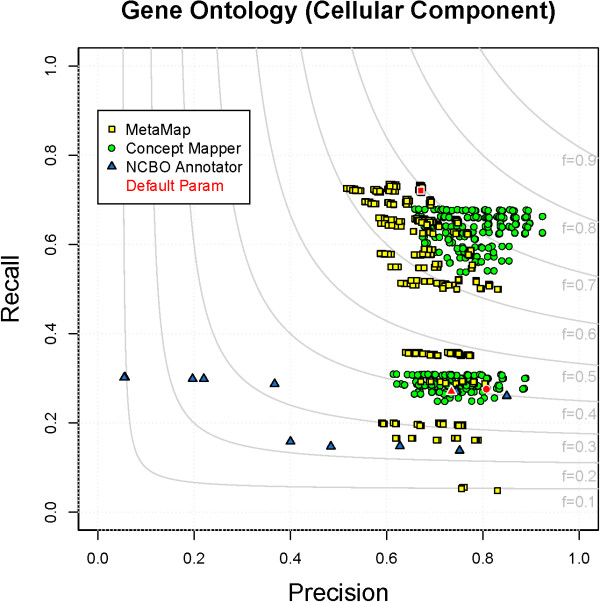
**All parameter combinations for GO_CC.** The distribution of all parameter combinations for each system on GO_CC. (MetaMap - yellow square, ConceptMapper - green circle, NCBO Annotator - blue triangle, default parameters - red).

The cellular component branch of the Gene Ontology describes locations at the levels of subcellular structures and macromolecular complexes. It is useful for annotating where gene products have been found to be localized. GO_CC is similar to CL in that it is a smaller ontology and contains very few synonyms, but the terms are slightly longer and more complex than CL (Table 1). Performance from CM (F = 0.77) is the best, with MM (F = 0.70) second, and NCBO Annotator (F = 0.40) third (Table 5). All parameter combinations for each system on GO_CC can be seen in Figure 3.

Just as in CL, there are many annotations to “GO:0005623 - cell”, 3,647 or 44% of all 8,354 annotations. We removed annotations of “cell” and saw a decrease in performance. Unlike CL, removal of these annotations does not affect all systems consistently. CM sees a large decrease in F-measure (0.2), while MM and NCBO Annotator see decreases of 0.08 and 0.02, respectively.

Precision for all parameter combinations of CM and MM are over 0.50, with the highest being CM at 0.92. NCBO Annotator widely varies from < 0.1 to 0.85. Because precision is high, there are very few FPs that are found. The FPs in common by all systems are due to less specific terms being found and ambiguous terms; NCBO Annotator also finds FPs from broad synonyms and MM specific errors are from abbreviations. Most of the common FPs are mentions that are less specific than the gold standard, due to higher-level terms contained within lower-level ones. For instance, “GO:0016020 - membrane” is found instead of a more specific type of membrane such as “vesicle membrane”, “plasma membrane”, or “cellular membrane”. All systems find ∼20 annotations of “GO:0042603 - capsule” when none are seen in CRAFT; this is due to overloaded terms from different biomedical domains. Because NCBO Annotator is a Web service, we have no control over versions of ontologies used, so it used a newer version of the ontology than that which was used to annotate CRAFT and as inputted into CM and MM. ∼42% of NCBO Annotator FPs were because “GO:0019814 - immunoglobulin complex, circulating” has a broad synonym “antibody” added. Because MM generates variants and incorporates synonyms, we see an interesting error produced from MM: “hair(s)” get annotated with “GO:0009289 - pilus”. It is not understandable why MM would assume this because “hair” is not a synonym, but in the GO definition, pilus is described as a “hair-like appendage”.

MM achieves the highest recall of 0.73 with CM slightly lower at 0.66 and NCBO Annotator the lowest (0.27). NCBO Annotator’s inability to recognize plurals and generate variants significantly hurts recall. NCBO Annotator can annotate neither “vesicles” with “GO:0031982 - vesicle” nor “autosomal” with “GO:0030849 - autosome”, which both CM and MM correctly annotate. The largest category of missed annotations represents other ways to refer to terms not in the synonym list. In CRAFT, “complex(es)” is annotated with “GO:0032991 - macromolecular complex”, and “antibody”, “antibodies”, “immune complex”, and “immunoglobulin” are all annotated with “GO:0019814 - immunoglobulin complex”, but no systems are able to identify these annotations because these synonyms do not exist in the ontology. MM achieves highest recall because it identifies abbreviations that other systems are unable to find. For example, “chr” is correctly annotated with “GO:0005694 - chromosome”, “ER” with “GO:0005783 - endoplasmic reticulum”, and “ECM” with “GO:0031012 - extracellular matrix”.

### Gene Ontology - Biological Process

**Figure 4:**
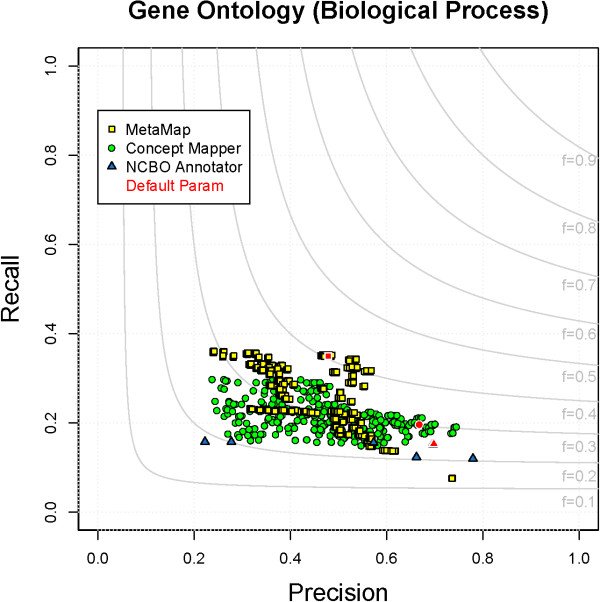
**All parameter combinations for GO_BP.** The distribution of all parameter combinations for each system on GO_BP. (MetaMap - yellow square, ConceptMapper - green circle, NCBO Annotator - blue triangle, default parameters - red).

Terms from GO_BP are complex; they have the longest average length, contain many words, and almost half contain stop words (Table 1). The longest annotations from GO_BP in CRAFT contain five tokens. Distribution of annotations broken down by number of words along with performance can be seen in Table 6. When dealing with longer and more complex terms, it is unlikely to see them expressed exactly in text as they are seen in the ontology. For these reasons, none of the systems performed very well. The maximum F-measures seen by each system can be seen in Table 5. All parameter combinations for each system on GO_BP can be seen in Figure 4. Examining mean F-measures for all parameter combinations, there is no difference in performance between CM (F = 0.37) and MM (F = 0.42), but considering only the top 25% of combinations there is a difference between the two. A statistical difference exists between NCBO Annotator (F = 0.25) and all others, under all comparison conditions.

**Table 6:** Word length in GO - Biological Process

**# Words**	**# CRAFT**	**% Found**	**% Found**	**% Found**
**in term**	**annotations**	**by CM**	**by MM**	**by NCBO**
5	7	14.3	14.3	14.3
4	109	17.4	3.7	9.2
3	317	37.2	33.4	35.0
2	2077	49.0	50.7	43.3
1	13574	27.6	34.2	11.6

Performance by all parameter combinations for all systems are grouped tightly along the dimension of recall. Precision for all systems is in the range of 0.2–0.8, with NCBO Annotator situated on the extremes of the range and CM/MM distributed throughout. Common categories of FPs encountered by all three systems are recognizing parts of longer/more specific terms and having different annotation guidelines. As seen in the previous ontologies, high-level terms are seen in lower level terms, which introduces errors in systems that find all matches. For example, we see NCBO Annotator incorrectly annotate “GO:001625 - death” within “cell death”, and both CM and MM annotate “development” with “GO:00032502 - developmental process” within the span “limb development”. Different annotation guidelines also cause errors to be introduced, e.g. all systems annotate “formation” with “GO:0009058 - biosynthetic process” because it has a synonym “formation”, but in CRAFT “formation” may be annotated with “GO:0032502 - developmental process”, “GO:0009058 - biosynthetic process”, or “GO:0022607 - cellular component assembly”, depending on the context. Most of the FPs common to both CM and MM are due to variant generation, for example, CM annotates “region(s)” with “GO:003002 - regionalization” and MM annotates “regular” and “regulator(s)” with “GO:0065007 - biological regulation”. Even though we see errors introduced through generating variants, many more correct annotations are produced.

In the grouping of all systems performance, recall lies between 0.1–0.4, which is low in comparison to most all other ontologies. More than ∼7,000 (> 50–60%) of the FNs are due to different ways to refer to terms not in the synonym list. The most missed annotation, with over 2,200 mentions, are those of “GO:0010467 - gene expression”; different surface variants seen in text are “expressed”, “express”, “expressing”, and “expression”. There are ∼800 discontiguous annotations that no systems are able to find. An example of a discontiguous annotation is seen in the following span: the textitd text from “*localization of* the Ptdsr *protein*” gets annotated with “GO:0008104 - protein localization”. Many of the annotations in CRAFT cannot be identified using the ontology alone so improvements in recall can be made by analyzing disparities between term name and the way they are expressed in text.

### Gene Ontology - Molecular Function

**Figure 5:**
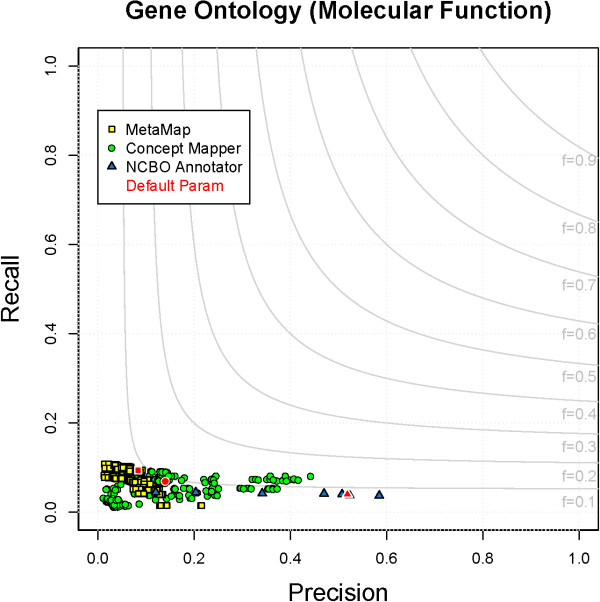
**All parameter combinations for GO_MF.** The distribution of all parameter combinations for each system on GO_MF. (MetaMap - yellow square, ConceptMapper - green circle, NCBO Annotator - blue triangle, default parameters - red).

The molecular function branch of the Gene Ontology describes molecular-level functionalities that gene products possess. It is useful in the protein function prediction field and serves as the standard way to describe functions of gene products. Like GO_BP, terms from GO_MF are complex, long, and contain numerous words with 52.8% containing punctuation and 26.6% containing numerals (Table 1). All parameter combinations for each system on GO_MF can be seen in Figure 5. Performance on GO_MF is poor; the highest F-measure seen is 0.14. Besides terms being complex, another nuance of GO_MF that makes their recognition in text difficult is the fact that nearly all terms, with the primary exception of binding terms, end in “activity”. This was done to differentiate the activity of a gene product from the gene product itself, for example, “nuclease activity” versus “nuclease”. However, the large majority of GO_MF annotations of terms other than those denoting binding are of mentions of gene products rather than their activities.

A majority of true positives found by all systems (> 70%) are binding terms such as “GO:0005488 - binding”, “GO:0003677 - DNA binding”, and “GO:0036094 - small molecule binding”. These terms are the easiest to find because they are short and do not end in “activity”. NCBO Annotator only finds binding terms while CM and MM are able to identify other types. CM identifies exact synonym matches; in particular, “FGFR” is correctly annotated with “GO:0005007 - fibroblast growth factor-activated receptor activity”, which has an exact synonym “FGFR”. MM correctly annotates “taste receptor” with “GO:0008527 - taste receptor activity”. These annotations are correctly found because the terms have synonyms that refer to the gene products as well as the activity. The only category of FPs seen between all systems is nested or less specific matches, but there are system-specific errors: NCBO Annotator finds activity terms that are incorrect, while MM finds many errors pertaining to synonyms. Example of incorrect nested annotations found by all systems are “GO:0005488 - binding” annotated within “transcription factor *binding*” and “GO:0016788 - esterase activity” within “acetylcholine *esterase*”. Because the CRAFT annotation guidelines purposely never included the term “activity”, some instances of annotating activity along with the preceding word is incorrect; for example, NCBO Annotator incorrectly annotates the span “recombinase activity” with “GO:0000150 - recombinase activity”. FPs seen only by MM are due to broad, narrow, and related synonyms. We see MM incorrectly annotate “neurotrophin” with “GO:0005165 - neurotrophin receptor binding” and “GO:0005163 nerve growth factor receptor binding” because both terms have “neurotrophin” as a narrow synonym.

Recall for GO_MF is low; at best only 10% of total annotations are found. Most of the annotations missed can be classified into three categories: activity terms, insufficient synonyms, and abbreviations. The category of activity terms is an overarching group that contains almost all of the annotations missed; we show performance can be improved significantly by ignoring the word activity in the next section. Terms that fall into the category of insufficient synonyms (∼30% of all terms not found) are not only missed because they are seen without “activity”. For instance, “hybridization(s)”, “hybridized”, “hybridizing”, and “annealing” in CRAFT are annotated with both “GO:0033592 - RNA strand annealing activity” and “GO:0000739 - DNA strand annealing activity”. These mentions are annotated as such because it is sometimes difficult to determine if the text is referring to DNA and/or RNA hybridization/annealing; thus, to simplify the task, these mentions are annotated with both terms, indicating ambiguity. Another example of insufficient synonyms is the inability of all systems to recognize “K + channel” as “GO:00005267 - potassium channel activity”, due to the fact that the former is not listed as a synonym of the latter in the ontology. A smaller category of terms missed are those due to abbreviations, some of which are mentioned earlier in the paper. For instance, in CRAFT, “Dhcr7” is annotated with “GO:0047598 - 7-dehydrocholesterol reductase activity” and “neo” is annotated with “GO:0008910 - kanamycin kinase activity”. Overall, there is much room for improvement in recall for GO_MF; ignoring “activity” at the end of terms during matching alone leads to an increase in R of 0.3.

#### Improving performance on GO_MF

**Figure 6:**
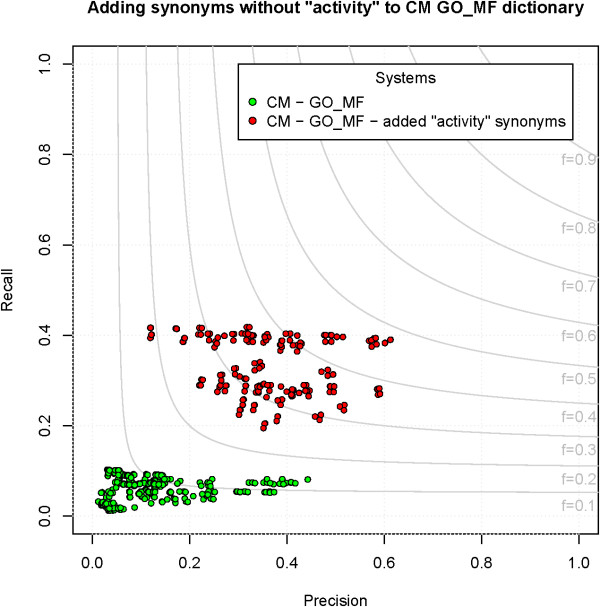
**Improvement seen by CM on GO_MF by adding synonyms to the dictionary.** By adding synonyms of terms without “activity” to the GO_MF dictionary precision and recall are increased.

As suggested in previous work on the GO, since the word “activity” is present in most terms, its information content is very low [33]. Also, when adding “activity” to the end of the top 20 most common other words in GO_MF terms (as seen in [51]), over half are terms themselves [52]. An experiment was performed to evaluate the impact of removing “activity” from all terms in GO_MF. For each term with “activity” in the name, a synonym was added to the ontology obo file with the token “activity” removed; for example, for “GO:0004872 - receptor activity”, a synonym of “receptor” was added. We tested this only with CM; the same evaluation pipeline was run but the new obo file used to create the dictionary. Using the new dictionary, F-measure is increased from 0.14 to 0.48 and a maximum recall of 0.42 is seen (Figure 6). These synonyms should not be added to the official ontology because it contradicts the specific guidelines the GO curators established [53], but should be added to dictionaries provided as input to concept recognition systems.

### Sequence Ontology

**Figure 7:**
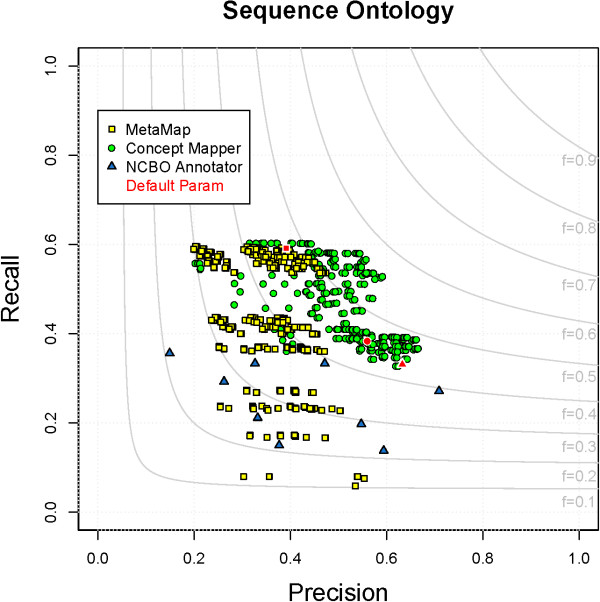
**All parameter combinations for SO.** The distribution of all parameter combinations for each system on SO. (MetaMap - yellow square, ConceptMapper - green circle, NCBO Annotator - blue triangle, default parameters - red).

The Sequence Ontology describes features and attributes of biological sequences. The SO is one of the smaller ontologies evaluated, ∼1,600 terms, but contains the highest number of annotations in CRAFT, ∼23,500. ∼92% of SO terms contain punctuation, which is due to the fact that the words of the primary labels are demarcated not by spaces but by underscores. Many, but not all, of the terms have an exact synonym identical to the official name, but with spaces instead of underscores. CM is the top performer (F = 0.56) with MM middle (F = 0.50) and NCBO Annotator at the bottom (F = 0.44). Statistically, looking at all parameter combinations mean F-measures, there is a difference between CM and the rest, while a difference cannot be determined between MM and NCBO Annotator. When looking at the top 25% of combinations, a difference can be seen between all three systems. All parameter combinations for each system on SO can be seen in Figure 7.

Most of the FPs can be grouped into four main categories: contextual dependence of SO, partial term matching, broad synonyms, and variants generated. In all three systems, we see the first three types, but errors from variants are specific to CM and MM. The SO is sequence specific, meaning that terms are to be understood in relation to biological sequences. When the ontology is separated from the domain, terms can become ambiguous. For example, “SO:0000984 - single” and “SO:0000985 - double” refer to the number of strands in a sequence, but can also be used in other contexts, obviously. Synonyms can also become ambiguous when taken out of context. For example, “SO:1000029 - chromosomal_deletion” has a synonym “deficiency”. In the biomedical literature, “deficiency” is commonly used when discussing lack of a protein, but as a synonym of “chromosomal_deletion” it refers to a deletion at the end of a chromosome; these are not semantically incorrect, but incorrect in terms of CRAFT concept annotation guidelines. Because of the hierarchical relationships in the ontology we find the high level term “SO:0000001 - region” within other terms; when the more specific terms are unable to be recognized, “region” can still be recognized. For instance, we find “region” incorrectly annotated inside the span “coding *region*”, when in the gold standard the span is annotated with “SO:0000851 - CDS_region”. Besides being ambiguous, synonyms can also be too broad. For instance, “SO:0001091 - non_covalent_binding_site” and “SO:0100018 - polypeptide_binding_motif” both have a synonym of “binding”; as seen in GO_MF above, there are many annotations of binding in CRAFT. The last category of errors are only seen in CM and MM because they are able to generate variants. Examples of erroneous variants are MM incorrectly annotating “based”, “foundation”, and “fundamental” with “SO:0001236 - base” and CM incorrectly annotating “probing” and “probed” with “SO:0000051 - probe”.

Recall on SO is close between CM (0.57) and MM (0.54), while recall for NCBO Annotator is 0.33. The ∼5,000 annotations found by both CM and MM that are missed by NCBO Annotator are composed of plurals and variants. The three categories that a majority of the FNs fall into are insufficient synonyms, abbreviations, and multi-span annotations. More than half of the FNs are due to insufficient synonyms or other ways to express a term. In CRAFT, “SO:0001059 - sequence_alteration” is annotated to “mutation(s)”, “mutant”, “alteration(s)”, “changes”, “modification”, and “variation”. It may not be the most intuitive annotation, but because of the structure of the SO version used in CRAFT, it is the most specific annotation that can be made for mutating/changing a sequence. Another example of insufficient synonyms can be seen from the annotation of “chromosomal region”, “chromosomal loci”, “locus on chromosome” and “chromosomal segment” with“SO:0000830 - chromosome_part”. These are more intuitive than the previous example; if different “parts” of a chromosome are explicitly enumerated the ability to find them increases. Abbreviations or symbols are another category missed. For example, “SO:0000817 - wild_type” can be expressed as “WT” or “+” and “SO:0000028 - base_pair” is commonly seen as “bp”. These abbreviations are more commonly seen in biomedical text than the longer terms are. There are also some multi-span annotations that no systems are able to find; for example, “*homologous* human MCOLN1 *region*” is annotated with “SO:0000853 - homologous_region”.

### Protein Ontology

**Figure 8:**
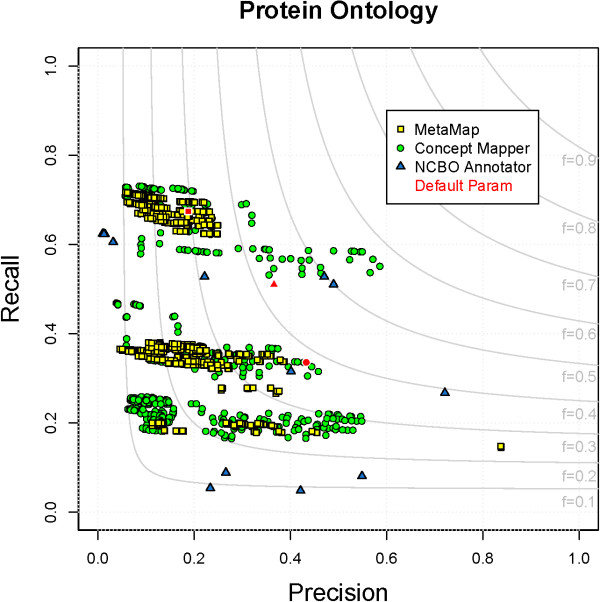
**All parameter combinations for PRO.** The distribution of all parameter combinations for each system on PRO. (MetaMap - yellow square, ConceptMapper - green circle, NCBO Annotator - blue triangle, default parameters - red).

The Protein Ontology (PRO) represents evolutionarily defined proteins and their natural variants. It is important to note that although the PRO technically represents proteins strictly, the terms of the PRO were used to annotate genes, transcripts, and proteins in CRAFT. Terms from PRO contain the most words, have the most synonyms, and ∼75% of terms contain numerals (Table 1). Even though term names are complex, in text, many gene and gene product references are expressed as abbreviations or short names. These references are mostly seen as synonyms in PRO. Recognizing and normalizing gene and gene product mentions is the first step in many natural language processing pipelines and is one of the most fundamental steps. CM produces the highest F-measure (0.57), followed by NCBO Annotator (0.50), and lastly MM (0.35) produces the lowest. All parameter combinations for each system on PRO can be seen in Figure 8. Unlike most of the ontologies covered above, stemming terms from PRO does not result in the highest performance. The best parameter combination for CM does not use any stemmer, which is why NCBO Annotator performs better than MM.

All systems are able to find some references to the long names of genes and gene products, such as “PR:000011459 - neurotrophin-3” and “PR:000004080 - annexin A7”. As stated previously, a majority of the annotations in CRAFT are short names of genes and gene products. For example, the long name of PR:000003573 is “ATP-binding cassette sub-family G member 8”, which is not seen, but the short name “Abcg8” is seen numerous times. The errors introduced by all systems can be grouped into misleading synonyms and different annotation guidelines, while MM also introduces errors from abbreviations and variants. Of errors common to all systems, the largest category is from misleading synonyms (> 50% for CM and NCBO Annotator, ∼33% for MM). For example, ∼3,000 incorrect annotations of “PR:000005054 - caspase-14”, which has synonym “MICE”, are seen, along with mentions of the word “male” incorrectly annotated with “PR:000023147 - maltose-binding periplasmic protein”, which has the synonym “malE”. As seen in these errors, capitalization is important when dealing with short names. Differing annotation guidelines also result in matching errors, but because all systems are at the same disadvantage a bias isn’t introduced. The word “protein” is only annotated with the ChEBI ontology term “protein”, but there are many mentions of the word “protein” incorrectly annotated with a high-level term of PRO, “PR:000000001 - protein”. This term was purposely not used to annotate “protein” and “proteins”, as this would have conflicted with the use of the terms of PRO to annotate not only proteins but also genes and transcripts. MM generates abbreviations and acronyms, but they are not always helpful. For example, due to abbreviations, “MTF-1” is incorrectly annotated with “PR:000008562 - histidine triad nucleotide-binding protein 2”; because MM is a black box, it is unclear how or why this abbreviation is generated. Morphological variants of synonyms are also causes of errors. For example, “finding” and “found” are incorrectly annotated because they are variants of “FIND”, which is a synonym of “PR:000016389 - transmembrane 7 superfamily member 4”.

All systems are able to achieve recall of >0.6 on at least one parameter combination, with CM and MM achieving 0.7 by sacrificing precision. When balancing P and R, the maximum R seen is from CM (0.57). Gene and gene product names are difficult to recognize because there is so much variation in the terms — not morphological variation as seen in most other ontologies, but differences in symbols, punctuation, and capitalization. The main categories of missed annotations are due to these differences. Symbols and Greek letters are a problem encountered many times when dealing with gene and gene product names [54]. These tools offer no translation between symbols so, for example, “TGF- *β*2” is unable to be annotated with “PR:000000183 - TGF-beta2” by any systems. Along the same lines, capitalization and punctuation are important. The hard part is knowing when and when not to ignore them; any of the FPs seen in the previous paragraph are found because capitalization is ignored. Both capitalization and punctuation must be ignored to correctly annotate the spans “mr-s” and “mrs” with “PR:000014441 - sterile alpha motif domain-containing protein 11”, which has a synonym “Mr-s”. As seen above, there are many ways to refer to a gene/gene product. In addition, an author can define one by any abbreviation desired and then refer to the protein in that way throughout the rest of the paper, so attempting to capture all variation in synonyms is a difficult task. In CRAFT, for instance, “snail” refers to “PR:000015308 - zinc finger protein SNAI1” and “moonshine” or “mon” refers to “PR:000016655 - E3 ubiquitin-protein ligase TRIM33”.

#### Removing FP PRO annotations

**Figure 9:**
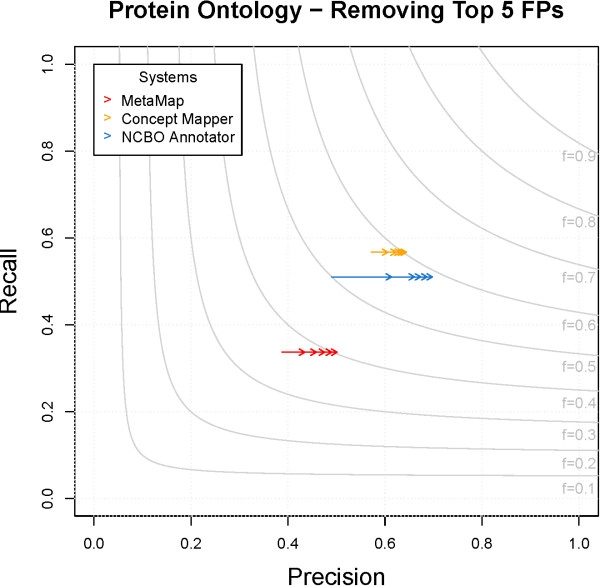
**Improvement on PRO when top 5 FPs are removed.** The top 5 FPs for each system are removed. Arrows show increase in precision when they are removed. No change in recall was seen.

In order to show that performance improvements can be made easily, we examined and removed the top five FPs from each system on PRO. The top five errors only affect precision and can be removed without any impact in recall; the impact can be seen in Figure 9. A simple process produces a change in F-measure of 0.03–0.09. A common category of FPs removed from all systems are annotations made with “PR:000000001 - protein”, as the term was found ∼1,000–3,500 times. Three out of the top five errors common to MM and NCBO Annotator were found because synonym capitalization was ignored. For example, “MICE” is a synonym of “PR:000005054 - caspase-14”, “FIND” is a synonym of “PR:000016389 - transmembrane 7 superfamily member 4”, and “AGE” is a synonym of “PR:000013884 - N-acylglucosamine 2-epimerase”. The second largest error seen in CM is from an ambiguous synonym: “PR:000012602 - gastricsin” has an exact synonym “PGC”; this specific protein is not seen in CRAFT, but the abbreviation “PGC” is seen ∼400 times referring to the protein peroxisome proliferator-activated receptor-gamma. By addressing just these few categories of FPs, we can increase the performance of all systems.

### NCBI Taxonomy

**Figure 10:**
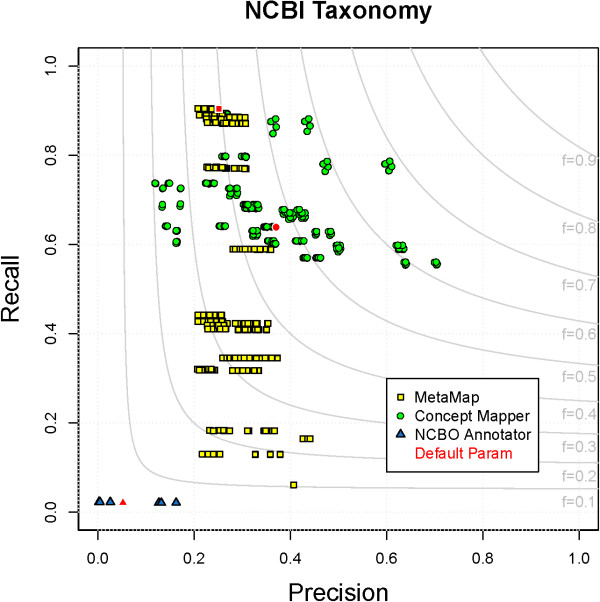
**All parameter combinations for NCBITaxon.** The distribution of all parameter combinations for each system on NCBITaxon. (MetaMap - yellow square, ConceptMapper - green circle, NCBO Annotator - blue triangle, default parameters - red).

The NCBI Taxonomy is a curated set of nomenclature and classification for all the organisms represented in the NCBI databases. It is by far the largest ontology evaluated, at almost 800,000 terms, but with only 7,820 total NCBITaxon annotations in CRAFT. Performance on NCBITaxon varies widely for each system: NCBO Annotator performs poorly (F = 0.04), MM performers better (F = 0.45) and CM performs best (F = 0.69). When looking at all parameter combinations for each system, there is generally a dimension (P or R) that varies widely among the systems and another that is more constrained (Figure 10).

In CRAFT, text is annotated with the most closely matching explicitly represented concept. For many organismal mentions, the closest match to an NCBI Taxonomy concept is a genus or higher-level taxon. For example, “mice” and “mouse” are annotated with the genus “NCBITaxon:10088 - Mus”. CM and MM both find mentions of “mice”, but NCBO Annotator does not. (Why will be discussed in the next paragraph.) All systems are able to find annotations to specific species; for example, “Takifugu rubripes” is correctly annotated with “NCBITaxon:31033 - Takifugu rubripes”. The FPs found by all systems are from ambiguous terms and terms that are too specific. Since the ontology is large and names of taxa are diverse, the overlap between terms in the ontology and common words in English and biomedical text introduces these ambiguous FPs. For example, “NCBITaxon:169495 - this” is a genus of flies, and “NCBITaxon:34205 - Iris germanica”, a species of monocots, has the common name “flag”. Throughout biomedical text there are many references to figures that are incorrectly annotated with “NCBITaxon:3493 - Ficus”, which has a common name of “figs”. A more biologically relevant example is “NCBITaxon:79338 - Codon” which is a genus of eudicots but also refers to a set of three adjacent nucleotides. Besides ambiguous terms, annotations are produced that are more specific than those in CRAFT. For example, “rat” in CRAFT is annotated at the genus level “NCBITaxon:10114 - Rattus”; while all systems incorrectly annotate “rat” with more specific terms such as, “NCBITaxon:10116 - Rattus norvegicus” and “NCBITaxon:10118 - Rattus sp.”. One way to reduce some of these false positives is to limit the domains in which matching is allowed, however, this assumes some previous knowledge of what the input will be.

Recall of > 0.9 is achieved by some parameter combinations of CM and MM, while the maximum F-measure combinations are lower (CM - R = 0.79 and MM - R = 0.88). NCBO Annotator produces very low recall (R = 0.02) and performs poorly due to a combination of: the way CRAFT is annotated and the way NCBO Annotator handles linking between ontologies. In NCBO Annotator, for example, the link between “mice” and “Mus” is not inferred directly, but goes through the MaHCO ontology [55], an ontology of major histocompatibility complexes. Because we limited NCBO Annotator to only using ontology directly tested, the link between “mice” and “Mus” is not used, and therefore are not found. For this reason, NCBO Annotator is unable to find many of the NCBITaxon annotations in CRAFT. On the other hand, CM and MM are able to find most annotations, the annotations missed are due to different annotation guidelines or specific species with a single-letter genus abbreviation. In CRAFT, there are ∼200 annotations of the ontology root, with text such as “individual” and “organism”; these are annotated because the root was interpreted as the foundational type of organism. An example of a single-letter genus abbreviation seen in CRAFT is “D. melanogaster” annotated with “NCBITaxon:7227 - Drosophila melanogaster”. These types of missed annotations are easy to correct for through some synonym management or post-processing step. Overall, most of the terms in NCBITaxon are able to be found and focus should be on increasing precision without losing recall.

### ChEBI

**Figure 11:**
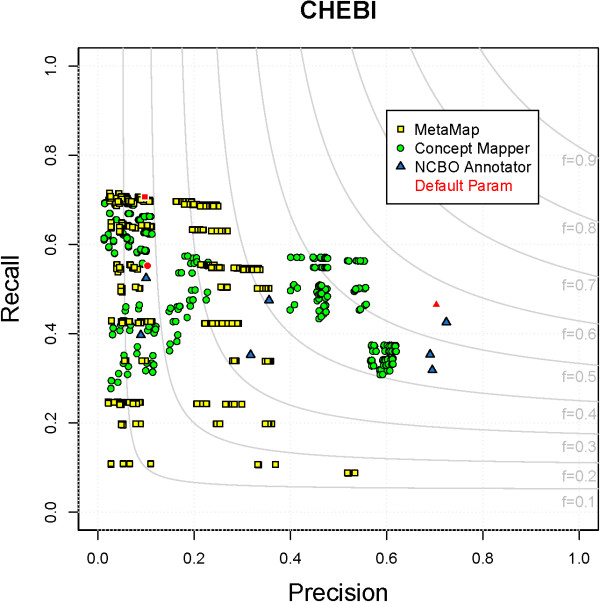
**All parameter combinations for ChEBI.** The distribution of all parameter combinations for each system on ChEBI. (MetaMap - yellow square, ConceptMapper - green circle, NCBO Annotator - blue triangle, default parameters - red).

The Chemical Entities of Biological Interest (ChEBI) Ontology focuses on the representation of molecular entities, molecular parts, atoms, subatomic particles, and biochemical rules and applications. The complexity of terms in ChEBI varies from the simple single-word compound “CHEBI:15377 - water” to very complex chemicals that contain numerals and punctuation, e.g., “CHEBI:37645 - luteolin 7-O-[(beta-D-glucosyluronic acid)-(1- >2)-(beta-D-glucosiduronic acid)] 4’-O-beta-D-glucosiduronic acid”. The maximum F-measure on ChEBI is produced by CM and NCBO Annotator (F = 0.56) with MM (F = 0.42) not performing as well. CM and MM both find ∼4,500 TPs, but because MM finds ∼5,000 more FPs its overall performance suffers (Table 4). All parameter combinations for each system on ChEBI can be seen in Figure 11.

There are many terms that all systems correctly find, such as “protein” with “CHEBI:36080 - protein” and “cholesterol” with “CHEBI:16113 - cholesterol”. Errors seen from all systems are due to differing annotation guidelines and ambiguous synonyms. Errors from both CM and MM come from generating variants while MM produces some unexplained errors. Different annotation guidelines lead to the introduction of both FPs and FNs. For example, in CRAFT, “nucleotide” is annotated with “CHEBI:25613 - nucleotidyl group”, but all systems incorrectly annotate “nucleotide” with “CHEBI:36976 - nucleotide” because they exactly match. (Mentions of “nucleotide(s)” that refer to nucleotides within nucleic acids are not annotated with “CHEBI:36976 - nucleotide” because this term specifically represents free nucleotides, not those as parts of nucleic acids.) Many FPs and FNs are produced by a single nested annotation; four gold-standard annotations are seen within “amino acid(s)”. Of these four annotations, two are found by all systems, “CHEBI:37527 - acid” and “CHEBI:46882 - amino”, while one introduces a FP: “CHEBI:33709 - amino acid” incorrectly annotated instead of “CHEBI:33708 - amino-acid residue”, while “CHEBI:32952 - amine” is not found by any system. Ambiguous synonyms also lead to errors; for example, “lead” is a common verb but also a synonym of “CHEBI:25016 - lead atom” and “CHEBI:27889 - lead(0)”. Variants generated by CM and MM do not always carry the same semantic meaning as the original term, such as “based” and “basis” from “CHEBI:22695 - base”. MM also produces some interesting unexplainable errors. For example, “disease” is incorrectly annotated with “CHEBI:25121 - maleoyl group”, “CHEBI:25122 - (Z)-3-carboxyprop-2-enoyl group”, and “CHEBI:15595 - malate(2-)”; all three terms have a synonym of “Mal”, but we could find no further explanations.

Recall for maximum F-measure combinations are in a similar range, 0.46–0.56. The two most common categories of annotations missed by all systems are abbreviations and a difference between terms and the way they are expressed in text. Many terms in ChEBI are more commonly seen as abbreviations or symbols. For instance, “CHEBI:29108 - calcium(2+)” is more commonly seen as “Ca2+”; even though it is a related synonym, the systems evaluated are unable to find it. A more complicated example can be seen when talking about the chemicals that lie on the ends of amino acid chains. In CRAFT, “C” from “*C*-terminus” is annotated with “CHEBI:46883 - carboxyl group” and “CHEBI:18245 - carboxylato group” (the double annotation indicating ambiguity among these), which all systems are unable to find; The same principle also applies for the N-terminus. One simple annotation that should be easy to get is “mRNA” annotated with “CHEBI:33699 - messenger RNA”, but CM and NCBO Annotator miss it. There is not always an overlap between the term names and their expression in text. For instance, the term “CHEBI:36357 - polyatomic entity” was chosen to annotate general “substance” words like “molecule(s)”, “substances”, and “compounds” and “CHEBI:33708 - amino-acid residue” is often expressed as “amino acid(s)” and “residue”.

An additional comparison between CM and ChemSpot [56], a ChEBI-specific named entity recognizer with machine learning components, on CRAFT can be seen in Additional file 3. The results of this comparison show that performance between both systems is similar and small changes to stop word lists and dictionaries can have a big impact of F-measure. It also explores FPs of ChemSpot, which could represent a potential missed annotation in CRAFT or lack of a ChEBI term for a chemical.

### Overall parameter analysis

Here we present overall trends seen from aggregating all parameter data over all ontologies and explore parameters that interact. Suggestions for parameters for any ontology based upon its characteristics are given. These observations are made from observing which parameter values and combinations produce the highest F-measures and not from statistical differences in mean F-measures.

#### NCBO Annotator

Of the six NCBO Annotator parameters evaluated, only three impact performance of the system: *wholeWordsOnly*, *withSynonyms*, and *minTermSize*. Two parameters, *filterNumber* and *stopWordsCaseSensitive*, did not impact recognition of any terms, while removing stop words only made a difference for one ontology (PRO).

A general rule for NCBO Annotator is that only whole words should be matched; matching whole words produced the highest F-measure on seven out of eight ontologies and on the eighth, the difference was negligible. Allowing NCBO Annotator to find terms that are not whole words greatly decreases precision while minimally, if at all, increasing recall.

Using synonyms of terms makes a significant difference in five ontologies. Synonyms are useful because they increase recall by introducing other ways to express concepts. It is generally better to use synonyms, as only one ontology performed better when not using synonyms (GO_MF).

*minTermSize* does not effect the matching of terms but acts as a filter to remove matches of less than a certain length. A safe value of *minTermSize* for any ontology would be one or three because only very small words (< 2 characters) are removed. Filtering terms less than length five is useful, not so much for finding desired terms, but for removing undesired terms. Undesired terms less than five characters can be introduced either through synonyms or small ambiguous terms that are commonly seen and should be removed to increase performance. (e.g. “NCBITaxon:3863 - Lens” and “NCBITaxon:169495 - This”)

#### Interacting parameters - NCBO Annotator

Because half of NCBO Annotator’s parameters do not affect performance, we only see interaction between two parameters: *wholeWordsOnly* and *synonyms*. The interactions between these parameters come from mixing *wholeWordsOnly* = no and *synonyms* = yes. As noted in the discussion of ontologies above, using this combination of parameters introduces anywhere from 1,000 to 41,000 FPs, depending on the test scenario and ontology. These errors are introduced because small synonyms or abbreviations are found within other words.

#### MetaMap

We evaluated seven MM parameters. The only parameter value that remained constant between all ontologies was *gaps*; we have come to the consensus that gaps between tokens should not be allowed when matching. By inserting gaps, precision is decreased with no or slight increase in recall.

The *model* parameter determines which type of filtering is applied to the terms. The difference between the two values for model is that strict performs an extra filtering step on the ontology terms. Performing this filtering increases precision with no change in recall for ChEBI and NCBITaxon with no differences between the parameter values on the other ontologies. Because it is best performing on two ontologies and in MM documentation is said to produce the highest level of accuracy, the strict model should be used for best performance.

One simple way to recognize more complex terms is to allow the reordering of tokens in the terms. Reordering tokens in terms helps MM to identify terms as long as they are syntactically or semantically the same. For example, “GO:0000805 - X chromosome” is equal to “chromosome X”. Practically, the previous example is an exception, as most reorderings are not syntactically or semantically similar; by ignoring token order, precision is decreased without an increase in recall. Retaining the order of tokens produces highest F-measure on six out of eight ontologies, while there was no difference on the other two. We conclude for best performance it is best to retain token order.

One unique feature of MM is that it is able to compute acronym and abbreviation variants when mapping text to the ontology. MM allows the use of all acronym/abbreviations (-a), only those with unique expressions (-u) and the default (no flags). For all ontologies, there is no difference between using the default or only those with unique expressions, but both are better than using all. Using all acronyms and abbreviations introduces many erroneous matches; precision is decreased without an increase in recall. For best performance, use default or unique values of acronyms and abbreviations.

Generating derivational variants helps to identify different forms of terms. The goal of generating variants is to increase recall without introducing ambiguous terms. This parameter produces the most varied results. There are three parameter values (all, none, and adj noun only), and each of them produces the highest F-measure on at least one ontology. Generating variants hurts the performance on half of the ontologies. Of these ontologies, variants of terms from PRO and ChEBI do not make sense because they do not follow typical English language rules while variants of terms in NCBITaxon and SO introduce many more errors than correct matches. all variants produce highest F-measure on CL, while adj noun only variants are best-performing on GO_BP. There is no difference between the three values for GO_CC and GO_MF. With these varied results, one can decide which type of variants to use by examining the way they expect terms in their ontology to be expressed. If most of the terms do not follow traditional English rules, like gene/protein names, chemicals, and taxa, it is best to not use any variants. For ontologies where terms could be expressed as nouns or verbs, a good choice would be to use the default value and generate adj noun only variants. This is suggested because it generates the most common types of variants, those between adjectives and nouns.

The parameters *minTermSize* and *scoreFilter* do not affect matching but act as a post-processing filter on annotations returned. *minTermSize* specifies the minimum length, in characters, of annotated text; text shorter than this is filtered out. This parameter acts exactly like that of the NCBO Annotator parameter with the same name presented above. MM produces scores in the range of 0 to 1000, with 1000 being the most confident. For all ontologies, a score of 1000 produces the highest P and the lowest R, while a score of 0 returns all matches and has the highest R with the lowest P, with 600 and 800 somewhere between. Performance is best on all ontologies when using most of the annotations found by MM, so a score of 0 or 600 is suggested. As input to MM, we provided the entire document; it is possible that different scores are produced when providing a phrase, sentence, or paragraph as input. The scores are not as important as the understanding that most of the annotations returned by MM are used.

#### ConceptMapper

We evaluated seven CM parameters. When examining best performance, all parameter values vary but one: *orderIndependentLookup* = off, which does not allow the reordering of tokens when matching, is set in the highest F-measure parameter combination for all ontologies. As for MM, it is best to retain ordering of tokens.

*searchStrategy* affects the way dictionary lookup is performed. contiguous matching returns the longest span of contiguous tokens, while the other two values (skip any match and skip any allow overlap) can skip tokens and differ on where the next lookup begins. Performance on six out of eight ontologies is best when only contiguous tokens are returned. On NCBITaxon, the behavior of *searchStrategy* is unclear and unintuitive: By returning non contiguous tokens, precision is increased without loss of recall. For most ontologies, only selecting contiguous tokens produces the best performance.

The *caseMatch* parameter tells CM how to handle capitalization. The best performance on four out of eight ontologies uses insensitive case matching while there is no difference between the values of *caseMatch* on three ontologies. There is no difference on those three because the best parameter combination utilizes the BioLemmatizer, which automatically ignores case. Thus, best performance on seven out of eight ontologies ignores case. PRO is the exception; its best-performing combination only ignores case on those tokens containing digits. For most ontologies, it is best to use insensitive matching.

Stemming and lemmatization allow matching of morphological term variants. Performance on only one ontology, PRO, is best when no morphological variants are used; this is the case because PRO terms annotated in CRAFT are mostly short names which do not behave and have the properties of normal English words. The best-performing combination on all other ontologies use either the Porter stemmer or the BioLemmatizer. For some ontologies, there is a difference between the two variant generators, and for others there was not. Even ontologies like ChEBI and NCBITaxon perform best with morphological variants because they are needed for CM to identify inflectional variants such as plurals. For most ontologies, morphological variants should be used.

CM can take a list of stop words to be ignored when matching. Performance on seven out of eight ontologies is better when stop words are not ignored. Ignoring PubMed stop words from these ontologies introduces errors without an increase in recall. An example of one error seen is the span “proteins that target” incorrectly annotated with “GO:0006605 - protein targeting”. The one ontology, NCBITaxon, where ignoring stop words results in best performance is due to a specific term, “NCBITaxon: 169495 - this”. By ignoring the word “this”, ∼1,800 FPs are prevented. If there is not a specific reason to ignore stop words, such as the terms seen in NCBITaxon, we suggest not ignoring stop words for any ontology.

By default CM only returns the longest match; all matches can be returned by setting *findAllMatches* to true. Seven out of eight ontologies perform better when only the longest match is returned. Returning all matches for these ontologies introduces errors because higher-level terms are found within lower-level ones and the CRAFT concept annotation guidelines specifically prohibit these types of nested annotations. CHEBI performs best when all matches are returned because it contains such nested annotations. If the goal is to find all possible annotations or it is known that there are nested annotations we suggest to set *findAllMatches* to true, but for most ontologies, only the longest match should be returned.

There are many different types of synonyms in ontologies. When creating the dictionary with the value all, all synonyms (exact, broad, narrow, related, etc...) are used; the value exact creates dictionaries with only the exact synonyms. The best performance on six out of eight ontologies uses only exact*synonyms*. On these ontologies, using only exact instead of all synonyms increases precision with no loss of recall; use of broad, related, and narrow synonyms mostly introduce errors. Performance on PRO and GO_BP is best when using all synonyms. On these two ontologies, the other types of synonyms are useful for recognition and increase recall. For most ontologies using only exact synonyms produces the best performance.

#### Interacting parameters - ConceptMapper

**Figure 12:**
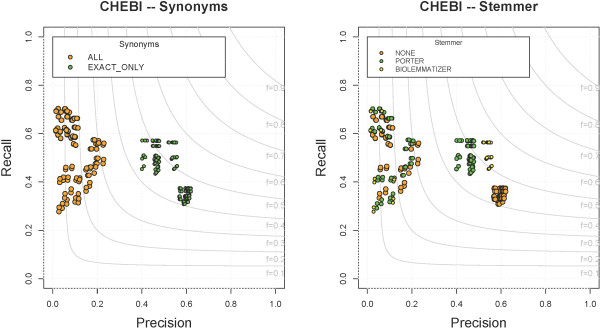
**Two CM parameter that interact on CHEBI.** Synonyms (left) and *stemmer* (right) parameter interact. The *stemmer* produce distinct clusters when only exact*synonyms* are used. When all*synonyms* are used, it is hard to distinguish any patterns in the *stemmer*.

We see the most interaction between parameters in CM. There are two different interactions that are apparent in certain ontologies: 1) *stemmer* and *synonyms* and 2) *stopWords* and *synonyms*. The first interaction found is in ChEBI. We find the *synonyms* parameter partitions the data into two distinct groups. Within each group, the stemmer parameter has two completely different patterns (Figure 12). When only exact*synonyms* are used all three stemmers are clustered, with BioLemmatizer performing best, but when all*synonyms* are used it is hard to find any difference between the three stemmers. The second interaction found is between the *stopWords* and *synonyms* parameters. In GO_MF several terms have synonyms that contain two words, with one being in the PubMed stop word list. For example, all mentions of “activity” are incorrectly annotated with “GO:0050501 - hyaluronan synthase activity”, which has a broad synonym “HAS activity”; “has” is contained in the stop word list and therefore is ignored.

Not only do we find interactions within CM, but some parameters also mask the effect of other parameters. It is already known and stated in the CM guidelines that the *searchStrategy* values skip any match and skip any allow overlap imply that *orderIndependentLookup* is set to true. Analyzing the data, it was also discovered that BioLemmatizer converts all tokens to lower case when lemmas are created, so the parameter *caseMatch* is effectively set to ignore. For these reasons, it is important to not only consider interactions but also the masking effect that a specific parameter value can have on another parameter.

### Substring matching and stemming

Through our analysis we have shown that accounting for morphology of ontological terms has an important impact on the performance of concept annotation in text. Normalizing morphological variants is one way to increase recall by reducing the variation between terms in an ontology and their natural expression in biomedical text. In NCBO Annotator, morphology can only be accommodated in the very rough manner of either requiring that ontology terms match whole (space or punctuation-delimited) words in the running text, or allowing any substring of the text whatsoever to match an ontology term. This leads to overall poorer performance by NCBO Annotator for most ontologies, through the introduction of large numbers of false positives. It should be noted that some substring annotations may appear to be valid matches, such as the annotation of the singular “cell” within “*cell*s”. However, given our evaluation strategy, such an annotation would be counted as incorrect since the beginning and end of the span do not directly match the boundaries of the gold CRAFT annotation. If a less strict comparator were used, these would be counted as correct, thus increasing recall, but many FPs would still be introduced through substring matching from e.g., short abbreviation strings matching many words.

MM always includes inflectional variants (plurals and tenses of verbs) and is able to include derivational variants (changing part of speech) through a configurable parameter. CM is able to ignore all variation (*stemmer* = none), only perform rough normalization by removing common word endings (*stemmer* = Porter), and handle inflectional variants (*stemmer* = BioLemmatizer). We currently do not have a domain-specific tool available for integration into CM to handle derivational morphology, as well, but a tool that could handle both inflectional and derivational morphology within CM would likely provide benefit in annotation of terms from certain ontologies. If NCBO Annotator were to handle at least plurals of terms, its recall on CL and GO_CC ontologies would greatly increase because many terms are expressed as plurals in text. For ontologies where terms do not adhere to traditional English rules (e.g.,ChEBI or PRO), using morphological normalization actually hinders performance.

### Tuning for precision or recall

**Figure 13:**
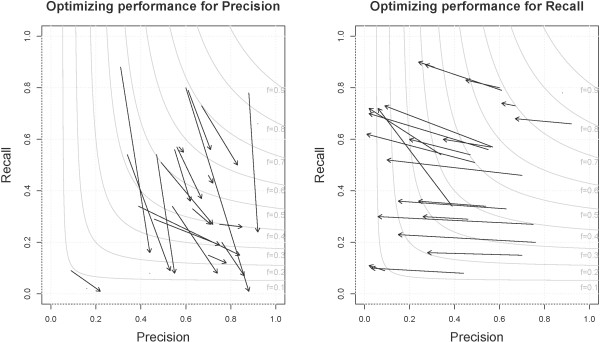
**Differences between maximum F-measure and performance when optimizing one dimension.** Arrows point from best performing F-measure combination to the best precision/recall parameter combination. All systems and all ontologies are shown.

We acknowledge that not all tasks require a balance between precision and recall; for some tasks high precision is more important than recall, while for others the priority is high recall and it is acceptable to sacrifice precision to obtain it. Since all the previous results are based upon maximum F-measure, in this section we briefly discuss the tradeoffs between precision and recall and the parameters that control it. The difference between the maximum F-measure parameter combination and performance optimized for either precision or recall for each system-ontology pair can be seen in Figure 13. By sacrificing recall, precision can be increased between 0 and 0.45. On the other hand, by sacrificing precision, recall can be increased between 0 and 0.38.

The best parameter combinations for optimizing performance for precision and recall can be seen in Table 7. Unlike the previous combinations seen above, parameters that produce the highest recall or precision do not vary widely between the different ontologies. To produce the highest precision, parameters that introduce any ambiguity are minimized; for example, word order should be maintained and stemmers should not be used. Likewise, to find as many matches as possible, the loosest parameter settings should be used; for example, all variants and different term combinations should be generated along with using all synonyms. The combination of parameters that produce the highest precision or recall are very different from the maximum F-measure-producing combinations.

**Table 7:** Best parameters for optimizing performance for precision or recall

**High precision annotations**					
**NCBO Annotator**		**MetaMap**		**ConceptMapper**	
**Parameter**	**Value**	**Parameter**	**Value**	**Parameter**	**Value**
wholeWordOnly	YES	model	STRICT	searchStrategy	CONTIGUOUS
filterNumber	ANY	gaps	NONE	caseMatch	SENSITIVE
stopWords	ANY	wordOrder	ORDER MATTERS	stemmer	NONE
SWCaseSensitive	ANY	acronymAbb	DEFAULT/UNIQUE	stopWords	NONE
minTermSize	THREE/FIVE	derivationalVariants	NONE	orderIndLookup	OFF
withSynonyms	NO	scoreFilter	1000	findAllMatches	NO
		minTermSize	3/5	synonyms	EXACT ONLY
**High recall annotations**					
**NCBO Annotator**		**MetaMap**		**ConceptMapper**	
**Parameter**	**Value**	**Parameter**	**Value**	**Parameter**	**Value**
wholeWordOnly	NO	model	RELAXED	searchStrategy	SKIP ANY/ALLOW
filterNumber	ANY	gaps	ALLOW	caseMatch	IGNORE/INSENSITIVE
stopWords	ANY	wordOrder	IGNORE	stemmer	Porter/BioLemmatizer
SWCaseSensitive	ANY	acronymAbb	ALL	stopWords	PubMed
minTermSize	ONE/THREE	derivationalVariants	ALL/ADJ NOUN	orderIndLookup	ON
withSynonyms	YES	scoreFilter	0	findAllMatches	YES
		minTermSize	1/3	synonyms	ALL

## Conclusions

After careful evaluation of three systems on eight ontologies, we can conclude that ConceptMapper is generally the best-performing system. CM produces the highest F-measure on seven out of eight total ontologies, while NCBO Annotator and MM both produce the highest F-measure on only one ontology (NCBO Annotator and MM produce equal F-measues on ChEBI). Out of all systems CM balances precision and recall the best; it produces the highest precision on four ontologies and the highest recall on three ontologies. The other systems perform well in one dimension but suffer in the other. MM produces the highest recall on five out of eight ontologies but precision suffers because it finds the most errors; the three ontologies for which it did not achieve highest recall are those where variants were found to be detrimental (SO, ChEBI, and PRO). On the other hand, NCBO Annotator produces the highest precision for four ontologies but falls behind in recall because it is unable to recognize plurals or variants of terms. Overall, CM performs best out of all systems evaluated on the concept normalization task.

Besides performance, another important thing to consider when using a tool is the ease of use. In order to use CM, one must adopt the UIMA framework. Transforming any ontology for matching is easy with CM with a simple tool that converts any OBO ontology file to a dictionary. MM is a standalone tool that works only with UMLS ontologies natively; getting it to work with any arbitrary ontology can be done but is not straightforward. MM is the most like a black box of all the systems, which results in some annotations that are unintuitive and cannot be traced to their source. NCBO Annotator is the easiest to use as it is provided as a Web service, with large retrieval occurring through a REST service. NCBO Annotator currently works with any of the 330+ BioPortal ontologies. Drawbacks of NCBO Annotator are due to it being provided as a Web service, they include changes in the underlying implementation, resulting in different annotations returned over time; there is also no control over the version of the ontologies used or the ability to add an ontology.

Using the default parameters for any tool is a common practice, but as seen here, the defaults often do not produce the best results. We have discovered that some parameters do not impact performance, while others greatly increase performance when compared to defaults. As seen in the Results and discussion Section, we have provided parameter suggestions for not only the ontologies evaluated but also provide general suggestions that can be applied to any ontology. We can also conclude that parameters cannot be optimized individually. If we didn’t generate all parameter combinations and instead examined parameters individually, we would be unable to see these interacting parameters and could have chosen a non-optimal parameter combination as the best.

Complex multi-token terms are seen in many ontologies and are more difficult to normalize than the simpler one- or two-token terms. Inserting gaps, skipping tokens, and reordering tokens are simple methods currently implemented in both CM and MM. These methods provide a simple heuristic but do not always produce valid syntactic structures or retain the semantic meaning of the original term. From our analysis above, we can conclude that more sophisticated, syntactically valid methods need to be implemented to recognize complex terms seen in ontologies such as GO_MF and GO_BP.

Our results demonstrate the important role of linguistic processing, in particular morphological normalization of terms, during matching. Several of the highest-performing sets of parameters take advantage of stemming or handling of morphological variants, though the exact best tool for this job is not yet entirely clear. In some cases, there is also an important interaction between this functionality and other system parameters, leading to some spurious results. It appears that these problems could be addressed in some cases through more careful integration of the tools and in others through simple adaptation of the tools to avoid some common errors that have occurred.

In this paper, we established baselines for performance of three publicly available dictionary-based tools on eight biomedical ontologies, analyzed the impact of parameters for each system, and made suggestions for parameter use on any ontology. We can conclude that of the tested tools, the generic ConceptMapper tool generally provides the best performance on the concept normalization task, despite not being specifically designed for use in the biomedical domain. The flexibility it provides in controlling precisely how terms are matched in text makes it possible to adapt it to the varying characteristics of different ontologies.

## Abbreviations

CM: ConceptMapper; MM: MetaMap; NCBO Annotator: NCBO Open Biomedical Annotator; TP: True positive; FP: False positive; FN: False negative; P: Precision; R: Recall; F: F-measure; GS: Gold standard; CL: Cell Type Ontology; GO_MF: Gene Ontology: Molecular Function; GO_CC: Gene Ontology: Cellular Component; GO_BP: Gene Ontology: Biological Process; ChEBI: Chemical Entities of Biological Interest; NCBITaxon: NCBI Taxonomy; SO: Sequence Ontology; PRO: Protein Ontology; Param: Parameter(s).

## Competing interests

The authors declare that they have no competing interests.

## Authors’ contributions

KV and LEH initiated the project and defined the overall research questions. KV, KBC, and CF planned the experiments. CF, WBJ, and CR constructed evaluation piplines and ran the experiments. CF and BG performed data and error analysis. CF wrote the first version of Methods, Results and discussion, and Conclusions Sections of the manuscript. KV, KBC, and CF wrote the Background and Introduction. MB contributed ontology expertise to the manuscript. KV, KBC, and LEH supervised all aspects of the work. All authors read and approved the final manuscript.

## Supplementary Material

Additional file 1 **Stop words list.** Text file that contains a list of stop words used. It consists of the PubMed stop words available at http://www.ncbi.nlm.nih.gov/books/NBK3827/table/pubmedhelp.T43/ along with the addition of the conjunction “or”. Click here for file

Additional file 2 **Detailed analysis of parameters for each system-ontology pair.** Here we present a detailed analysis of the statistically significant parameters for each system-ontology pair. Many interesting examples are given to show the impact of changing a single parameter. Click here for file

Additional file 3 **Comparison of CM to ChemSpot.** Comparison between ConceptMapper and ChemSpot, a ChEBI specific named entity recognition tool. It was performed and written up as a lab rotation by Benjamin Garcia. Click here for file
