# Supplementary material for: Large-scale biomedical concept recognition: an evaluation of current automatic annotators and their parameters
Source: BMC Bioinformatics. 2014 Feb 26;15:59. doi: 10.1186/1471-2105-15-59 (PMC4015610; doi:10.1186/1471-2105-15-59)
Supplement: Additional file 2 — Detailed analysis of parameters for each system-ontology pair. Here we present a detailed analysis of the statistically significant parameters for each system-ontology pair. Many interesting examples are given to show the impact of changing a single parameter. [file 1471-2105-15-59-S2.pdf]

Additional File 2 - Detailed analysis of parameters for each system-ontology pair

This additional file presents the more detailed analysis (steps 2 and 3) not covered in the main manuscript.

1. At the ontology level. This provides a synopsis of overall performance for each system with comments about common terms correct (TP), errors (FP), and categories missed (FN). Specific examples are taken from the top performing, highest F-measure parameter combination.

2. A high-level parameter analysis, performed over all parameter combinations. This allows for observation about impact on performance seen by manipulating parameter values, presented as ranges of impact.

3. A low-level analysis obtained from examining individual result files gives insight into specific terms or categories of terms that are affected by manipulating parameters.

Cell Type Ontology

NCBO Annotator parameters - CL

Two parameters were found to be statistically significant: *wholeWordsOnly* ($p=2.24 \times 10^{-6}$) and *minTermSize* ($p=9.68 \times 10^{-15}$). By using *wholeWordsOnly* = *yes* precision is increased 0.6-0.7 with no change in recall; because of low recall, F-measure is only increased by 0-0.1. Allowing matching to non-whole words finds ~100 more correct annotations but also ~7500 more FPs. Correct annotations found are mostly due to hyphenated spans: "one-neuron" is correctly annotated with "CL:0000540: neuron" and "fibroblast-like" with "CL:0000057 - fibroblast". About half of the FPs found are due to plurals, "cell" is found within "cells" and "cellular", while "neuron" is found within "neuronal" and "neurons". There are FPs because the beginning and end of the text span do not match exactly. Synonyms can be misleading when mixed with matching non-whole words. "CL:0000502 - type D enteroendocrine cell" has a synonym "D cell", which is found in the following spans "shaped cells", "elongated cells", "Disorganized cellular". Correct terms found do not outweigh the errors introduced, so it is most effective to restrict matching to only whole words.

There is no difference between filtering terms of size *one* or *three* characters. When filtering out terms less than *five* characters, recall drops 0.15 and F-measure decreases 0.1-0.2. Since "cell" is less than five characters and makes up a large proportion of the annotations, it is understandable why a large decrease in performance is seen. It is best to only filter smaller terms, less than *one* or *three* characters.

MetaMap parameters - CL

Three MM parameters were found to be statistically significant: *scoreFilter* ($p=2.2 \times 10^{-16}$), *minTermSize* ($p=1.1 \times 10^{-9}$), and *gaps* ($p=8.9 \times 10^{-8}$). *scoreFilter* and *minTermSize* act as a filter on terms and do not effect the way matching is performed. The best F-measures on CL do not filter any terms, so a score of 0 is suggested. For the same reasons as the NCBO Annotator parameter *minTermSize* seen above, *one* and *three* are best for filtering term length.

The *gaps* parameter allows skipping tokens to facilitate matching. Allowing gaps increases R <0.05 and decreases P 0-0.2, with a loss of 0.1 in F. Allowing gaps proved useful, and found ~200 more correct terms. We found words of some term names were not needed to fully express meaning: “apoptotic cell(s)” is correctly annotated with “CL:0000445 - apoptosis fated cell”. Allowing gaps also introduces ~1200 more incorrect annotations. Terms that are less specific than the annotations seen in CRAFT are incorrectly recognized, such as: “hair cell(s)” being annotated with “CL:0000346 - hair follicle dermal papilla cell”. Mixing abbreviations and gaps produced an interesting incorrect result: “ES cell(s)” is now annotated with “CL:0000715 - embryonic crystal cell”. Due to such introduced errors, although allowing gaps found an increased number of correct terms, the overall F-measure decreased.

Concept Mapper parameters - CL

Four CM parameters were found to be statistically significant: *stemmer* ($p=2.2 \times 10^{-16}$), *orderIndependent-Lookup* ($p=3.26 \times 10^{-7}$), *searchStrategy* ($p=2.2 \times 10^{-16}$), and *synonyms* ($p=5.24 \times 10^{-3}$). It is not conclusive as to which stemmer to use (Porter or BioLemmatizer), but the fact is clear that a stemmer should be used. Not using a stemmer decreases recall by 0.6 with no change in precision. Using a stemmer allows “neuron” to be converted to “neuronal”, “apoptosis” to be found as “apoptotic”, and most importantly allows plurals to be found. Without a stemmer, performance is very similar to the NCBO Annotator.

The parameter *searchStrategy* controls the way possible matches are found in the dictionary. *contiguous* produces highest performance, with an increase in R of 0.1-0.3 and an increase in P of 0.2-0.4 over the other values. Allowing CM to ignore tokens converts correct annotations into incorrect annotations because the span length increases to encompass more tokens. Using *contiguous* matching, the span “hair cells” is correctly annotated with “CL:0000855 - sensory hair cell” but when *skip any match* is used we see a completely different annotation, “cochlear immature hair cells” is incorrectly annotated with “CL:0000202 - auditory hair cell”. It should be noted that this is considered to be an incorrect annotation in our analysis. By employing a comparator that takes into account the hierarchy of the ontology, this could be given partial

credit since "sensory hair cell" is a parent of "auditory hair cell" (Verspoor *et al* 2006) We can also get complete nonsense when mixing *synonyms* and *searchStrategy*: "CL:0000570 - parafollicular cell" has a synonym "C cell" which gets annotated to "C) Positive control showing granule cells" and masks the correct annotation of "CL:0000120 - granule cell".

When using *exact synonyms* over *all* we see an increase in P of 0.1-0.15 with no change in R. Using *all synonyms*, ~400 more incorrect annotations are produced; broad synonyms are the cause. "CL:0000562 - nucleate erythrocyte" and "CL:0000595 - enucleate erythrocyte" both have broad synonyms of "red blood cell" and "RBC", which are found many times. Also, "CL:0000560 - band form neutrophil" has a broad synonym "band" which is not specific; it could be talking about a band on a gel or referring to muscle. Because CL doesn't contain many synonyms, the meaning is contained well enough to only use *exact synonyms*.

Gene Ontology - Cellular Component
NCBO Annotator parameters - GO_CC

Two parameters were found to be significant: *minTermSize* ($p=5.9 \times 10^{-8}$) and *wholeWordsOnly* ($p=3.7 \times 10^{-9}$). Given that 44% of annotations in CRAFT are "cell", filtering terms less than *five* characters removes many correct annotations. There is no difference between filtering *one* and *three* length terms. If using the combination of *wholeWordsOnly = no* and *synonyms = yes*, which we do not recommend, it is better to filter terms less than *three*. "GO:0005783 - endoplasmic reticulum" has a synonym "ER" which is a common combination of letters seen ~32,500 times in CRAFT in words such as "oth<u>er</u>", "w<u>er</u>e", "exp<u>er</u>iments", and "promot<u>er</u>". Using *wholeWordsOnly = no* allows NCBO Annotator to find ~250 more correct annotations (e.g. "<u>membrane</u>-bound" is correctly annotated with "GO:0016020 - membrane" and "<u>collagen</u>-related" with "GO:0005581 - collagen"), but it also finds ~41,000 more false positives (including erroneous annotations of "er"). Examples of incorrect terms found that are not due to synonyms are plurals, "<u>vesicle</u>s" incorrectly annotated with "GO:0031982 - vesicle". These are counted as errors because we used a strict comparator, where beginning and end of the text span must match. If a more lenient comparator were used, these types of errors would be considered correct.

MetaMap parameters - GO_CC

Three parameters were found to be statistically significant: *acronymAbb* ($p=1.2 \times 10^{-5}$), *scoreFilter* ($p=2.2 \times 10^{-16}$), and *minTermSize* ($p=1.4 \times 10^{-11}$). MM offers multiple ways to compute and use acronyms or

abbreviations to help resolve ambiguous terms. We find it best to use the parameter values *default* or *unique*. The other value, *all*, uses all acronyms or abbreviations. When using *all* instead of *unique*, we see a decrease in P of 0.05-0.2 and slight decrease in R; ~80 less TPs and ~1,500 more FPs are found by the maximum F-measure parameter combination. It is unclear why using *all acronymAbb* finds fewer correct annotations than using only those with *unique* expansions. The annotations missed appear to have nothing to do with acronyms or abbreviations but actually derivations. Examples of annotations that were missed by using *all* instead of *unique* are "cytoplasmic" annotated with "GO:0005737 - cytoplasm" and "cytoskeletal" annotated with "GO:0005856 - cytoskeleton". Errors introduced by using *all* do look like they came from acronyms or abbreviations. For example, "lung(s)", "pulmonary artery", "pulmonary", "pathological", and "pathology" are all incorrectly annotated with "GO:0000407 - pre-autophagosomal structure", which has a synonym "PAS". "PAS" is an abbreviation for "periodic acid-schiff", a staining method commonly used to stain glycoproteins in the lungs, but it is unlikely that MM makes this linked logical jump; it is unclear why these terms get annotated. It is best to use *default* or *unique* for *acronymAbb*.

It is best to not filter out many terms and use a *scoreFilter* of 0 or 600, because R decreases 0.2-0.6 when using a score of 800 or 1000. Just like the NCBO Annotator parameter examined above, filtering terms less than 5 characters removes many correct annotations of "cell"; it is best to filter less than 1 or 3.

Concept Mapper parameters - GO_CC

Four parameters were found to be statistically significant: *searchStrategy* ($p=2.2 \times 10^{-16}$), *stemmer* ($p=2.2 \times 10^{-16}$), *findAllMatches* ($p=4.8 \times 10^{-5}$), and *synonyms* ($p=1.3 \times 10^{-4}$). The *searchStrategy contiguous* produces the best performance; we see an increase in P of 0.1 over *skip any allow overlap* and increase in both P of 0.1 and R of 0.05 over *skip any match*. Using any other *searchStrategy* besides *contiguous* allows correct annotations to be masked by inclusion of surrounding tokens. The span "chromatin granules and fusion of membranes" is incorrectly annotated with "GO:0042584 - chromatin granule membrane" when using *skip any match*, but the underlined sub-span is correctly annotated with "GO:0042583 - chromatin granule" when using *contiguous* matching.

It is significantly better to use a stemmer, which results in an increase in P of 0.1-0.2 and R of 0.4. It is not clear which stemmer is better. Since both stemmers have similar performance, we will only discuss one. Using Porter over *none* introduces ~3,000 correct annotations while only finding ~150 errors. Plurals and variants such as "chromosomal" and "chromosomes" are correctly annotated with "GO:0005694 - chromosome" and "cytoplasmic" correctly annotated with "GO:0005737 - cytoplasm". Not all variants generated by stemming

are valid, for example, “fibrillate(s)” and “fibrillation” get annotated with “GO:0043205 - fibril”. Overall, the majority of variants are helpful.

Creating dictionaries using *all synonyms* instead of *exact* decreases P 0.05 with no loss of R. Broad synonyms are the source of these errors; “GO:0035003 - subapical complex” has a broad synonym of “SAC” which is seen ~100 times in PMID 17608565 as an abbreviation for “starburst amacrine cells”. “GO:0019013 - viral nucleocapsid” has a broad synonym of “core” which is found numerous times throughout CRAFT not referring to anything viral. Like CL, there are very few synonyms in GO_CC and we can conclude other types of synonyms are not used frequently in text.

Gene Ontology - Biological Process

NCBO Annotator parameters - GO_BP

Only one parameter was found to be significant, *wholeWordsOnly* (p=1.33 $\times 10^{-7}$). Allowing NCBO Annotator to match non-whole words, only ~70 more correct annotations found while allowing ~6000 more incorrect matches, resulting in a decrease in P of 0.1-0.5 with a small increase in R. Correct annotations found are due to hyphenated text, for example, “gene expression” from the span “target-gene expression” and “one-gene expression” are correctly annotated with “GO:0010467 - gene expression”. A few FP found are from finding whole terms within other words in the text, e.g. “GO:0007618 - mating” found within “estimating”. Using synonyms with matching of non-whole words introduces the majority of errors seen. For instance, “GO:0031028 - septation ignition signaling cascade” has an exact synonym “SIN”, which is found ~2200 times in words such as “using”, “single”, “increasingly”, and “encompassing”. We suggest using *wholeWordsOnly = yes* for maximum F-measure and P.

MetaMap parameters - GO_BP

Three parameters were found to be significant: *gaps* (p=1.8 $\times 10^{-6}$), *derivationalVariants* (p=2.8 $\times 10^{-10}$), and *scoreFilter* (p=2.2 $\times 10^{-16}$). One way to approximate variation in complex terms is to allow MM to skip tokens to find a match. By allowing gaps, ~75 more TPs are found but the solution isn’t optimal because ~7,500 more FPs are also found; P decreases 0.2-0.3 with a small increase in R. Skipping tokens helps correctly annotate “photoreceptor morphogenesis” with “GO:0008594 - photoreceptor cell morphogenesis” and “meiotic checkpoint” with “GO:0033313 - meiotic cell cycle checkpoint”, but because of the structure of terms in GO_BP we see many more errors. Many terms share similar token patters and by allowing MM to skip tokens many incorrect annotations are produced. For example “regulated process” is incorrectly anno-

tated with 193 different GO terms, such as "GO:0009889 - regulation of biosynthetic process", "GO:0042053 - regulation of dopamine metabolic process", and "GO:0045363 - regulation of interleukin-11 biosynthetic process".

Another way to help find variants of terms in text is to use derivational variants. It is best to generate variants, but there is no significant difference between which type of variants, *all* or *adj noun only*. Generating variants trades precision for recall. When comparing *none* to *adj noun only*, we see an increase in R of 0.05-0.2 along with a decrease in P of 0-0.1. For the best parameter combination, ~2,000 more TPs are found along with ~1,700 more FPs. Not using variants correctly annotates "development" with "GO:0032502 - developmental process" but when adding *adj noun only* variants, "developmental", "developmentally", "developing", and "develop(s)" are also correctly annotated. Generating variants does not always produce semantically similar terms because ambiguities are introduced. For example, "GO:0007586 - digestion" refers to the process of breaking down nutrients into components that are easily absorbed, but variants such as "digestion(s)", "digested", and "digesting" also refer to the process of fragmenting DNA using enzymes. Even though errors are introduced, it is still best to generate variants of terms.

Concept Mapper parameters - GO_BP

Four parameters were found to be statistically significant: *searchStrategy* (p=2.2 $\times 10^{-16}$), *orderIndependentLookup* (p=9.8 $\times 10^{-11}$), *findAllMatches* (p=4.0 $\times 10^{-10}$), and *synonyms* (p=2.4 $\times 10^{-9}$). Like MM, CM also has the ability to approach matching of complex terms through the use of *searchStrategy* and *orderIndependentLookup*. Setting CM's *searchStrategy* = *skip any match* we see ~10 more correct annotations found while allowing ~3,000 more incorrect ones to be found. This can be seen when CM correctly annotates "DNA damage repair" with "GO:0006821 - DNA repair" but also incorrectly annotates the long span "photoreceptors were significantly altered in the their expression level in the Crx-/- mouse, there are many candidates that could be important for photoreceptor morphogenesis" with "GO:'0046531 - photoreceptor cell development". It is interesting to note that the correct and incorrect annotations found by changing MM's *gaps* parameter are not seen when making the similar change in CM and vice versa; even though the parameter should have the same effect on matching, the same annotations are not produced because the systems have different underlying methods.

Besides skipping tokens, another way to approach complex terms is to allow token reordering. Allowing CM to reorder tokens decreases P 0-0.1 with varying small impact on R. In the case of the maximum F-measure parameter combination, varying token order only allows 1 more TP to be found but ~200 more FPs.

Word reordering only helped to find "endoplasmic reticulum protein retention" annotated with "GO:0006621 - protein retention in ER lumen". Finding that single term also introduces errors such as "activated cell(s)" incorrectly annotated with "GO:0001775 - cell activation" and "of apoptosis induction" incorrectly annotated with "GO:0006917 - induction of apoptosis". The benefits of finding the single correct term do not outweigh the errors also introduced; it is best to not allow reordering of tokens for GO_BP.

Stemming is useful for accounting for variations between terms in the ontology and their morphological variations see in text. Using Porter instead of BioLemmatizer or *none*, precision is traded for recall, but a higher F-measure is produced. Comparing Porter to *none*, ~1,300 more TPs are found, but also ~3,500 more FPs are found. CM with Porter, for example, correctly annotates "regulate", "regulating", and "regulated" with "GO:0065007 - biological regulation" and "transcriptional" with "GO:0006351 - transcription, DNA-dependent". Some of the incorrect annotations seen are "transcript(s)" annotated with "GO:0006351 - transcription, DNA-dependent" and "signal(s)" annotated with "GO:0007165 - signal transduction". It is interesting to see that for the single ontology term, "transcription DNA-dependent", both TPs and FPs can be generated by changing the endings.

Gene Ontology - Molecular Function

NCBO Annotator parameters - GO_MF

The only parameter found to be statistically significant is *wholeWordsOnly* (p=1.82 $\times 10^{-6}$). Since most of the correct annotations found are related to binding, one can imagine that allowing to match non-whole words leads to many incorrect instances of "GO:0005488 - binding" being found. When allowing matching to non-whole words, precision decreases 0.1-0.4. Even though we see a decrease in P, F-measure is only decreased 0.01 because R is so low. ~20 more TPs are found within hyphenated text, e.g. "substrate-binding" is correctly annotated with "GO:0005488 - binding". But not all hyphenated nested terms are correct. ~70 more errors are also introduced; for instance, "phospholipid-binding" is incorrectly annotated with "GO:005488 - binding". We also see full terms within other words, "GO:0003774 - motor activity" is incorrectly annotated within "locomotor activity". It is difficult to provide suggestions because the highest mean F-measures, 0.075, are obtained by using *wholeWordsOnly* = *no*, but using *wholeWordsOnly* = *yes* produces a mean F-measure of 0.070. There is a statistically significant difference between the two, but practically speaking they are both poor.

MetaMap parameters - GO_MF

Four parameters were found to be statistically significant: *gaps* (p=2.2 $\times 10^{-16}$), *acronymAbb* (p=5.2 $\times 10^{-5}$), *scoreFilter* (p=2.2 $\times 10^{-16}$), and *minTermSize* (p=1.6 $\times 10^{-9}$). Even though these four parameters produce statistically significant mean F-measures, it is difficult to analyze them because for most parameter combinations P, R, and F are all less than 0.1. The *gaps* parameter shows the biggest difference between F-measure in parameter values, 0.02. Allowing gaps introduces ~10 more correct annotations along with ~4,000 more incorrect ones. Of the few correct annotations found by allowing gaps, one example is, "Ran-binding" correctly annotated with "GO:0008536 - Ran GTPase binding". The errors introduced from allowing gaps are due to similarities in terms in the ontology. For instance, "D activity" is incorrectly annotated with 170 different GO terms, such as, "GO:0047816 - D-arabinose 1-dehydrogenase activity" and "GO:00428880 - D-glucuronate transmembrane transporter activity". For best performance, gaps should not be allowed.

scoreFilter and *minTermSize* are filters on the returned annotations and do not affect the way matching is performed. The maximum F-measures are seen when *scoreFilter* is set to 0 or 600 and *minTermSize* is set to 1 or 3. These parameter settings return most of the annotations found by MM.

Concept Mapper parameters - GO_MF

Four parameters were found to be statistically significant: *searchStrategy* (p=2.2 $\times 10^{-16}$), *stopWords* (p=5.8 $\times 10^{-13}$), *findAllMatches* (p=2.2 $\times 10^{-16}$), and *synonyms* (p=4.3 $\times 10^{-16}$). Using *contiguous* *searchStrategy* produces the highest F-measure; an increase in P of 0.05-0.3 and an increase in R of 0-0.05 is seen when comparing to other values. Allowing CM to skip tokens when looking terms up converts TPs to FPs because more tokens are included. For example, using *contiguous*, "GO:0005488 - binding" is correctly annotated in the span "proteins that bind", but when using *skip any match*, the same span is incorrectly annotated with "GO:0005515 - protein binding". We see an interaction between *searchStrategy* and *findAllMatches*. When using a value of *searchStrategy* that allows gaps along with *findAllMatches* = *yes*, recall is increased and a higher F-measure is seen.

It is recommended to not remove stop words; both P and R are decreased when removing PubMed stop words. ~15 TPs found when not removing stop words are missed when removing PubMed stop words because more specific annotations can be made by ignoring a common word. For example, when stop words are not removed "bind" is correctly annotated with "GO:0005488 - binding", but when removing PubMed stop words, other binding annotations are produced from the same span, such as, "proteins that bind" is incorrectly annotated with "GO:0005515 - protein binding" and "receptors, which bind" is incorrectly

annotated with "GO:0005102 - receptor binding". Besides the missed annotations seen above, ~1,000 more errors are introduced. Most of these errors are from synonyms with the *caseMatch* parameter set to *ignore* or *insensitive*. For instance, "bind(s)", "binding", "bound" are incorrectly annotated with "GO:0003680 - AT DNA binding", which has exact synonym "AT binding", which contains a stop word. Along the same lines, "activity" is incorrectly annotated with "GO:0050501 - hyaluronan synthase activity", which has a broad synonym "HAS activity", where "has" is a stop word.

Creating dictionaries with *all synonyms* introduces ~100 more TPs and ~11,000 more FP. Using narrow synonyms helps to correctly identify "ryanodine receptor" with "GO:0005219 - ryanodine-sensitive calcium-release channel activity". But overall, using *all synonyms* hurts performance. Related synonyms for some terms are common words. For example, "GO:0004066 - asparagine synthesis activity" has a related synonym "as", which is found more than 2,000 times in CRAFT. We also see many interesting errors introduced when mixing a stemmer and all synonyms. "GO:0043807 - 3-methyl-2-oxobutanoate dehydrogenase (ferredoxin) activity" has a related synonym "VOR", which when run though BioLemmatizer produces the lemma "for" and is found over 4,000 times in CRAFT. We suggest using *exact* synonyms.

Sequence Ontology

NCBO Annotator parameters - SO

Two parameters were found to be significant: *wholeWordsOnly* ($p=8.6 \times 10^{-11}$) and *minTermSize* ($p=2.5 \times 10^{-5}$). Allowing NCBO Annotator to match non-whole words introduces ~500 more correct annotations, but as a result, ~40,000 more incorrect ones are also found resulting in a decrease in P of 0.2-0.4 with a small decrease in R. Correct annotations found are from hyphenated spans of text. For example, "SO:0001026 - genome" is correctly found within "genome-wide", and "GO:0000704 - gene" is also correctly found within "gene-based" and "receptor-gene". Many errors are introduced given the ability to recognize non-whole words. Smaller terms are found within other words. "SO:0000704 - gene"; for example, is found within "morphogeneic", "general", and "degenerate".

Filtering terms less than *five* characters decreases R by 0.2. This is due to the fact that two commonly found correct annotations will be filtered out, "SO:0000704 - gene" and "SO:0000352 - DNA". For best performance, terms less then length *one* or *three* should be filtered.

MetaMap parameters - SO

Four parameters were found to be different: *model* (p=1.6 $\times 10^{-6}$), *acronymAbb* (p=2.8 $\times 10^{-9}$), *scoreFilter* (p=2.2 $\times 10^{-16}$) and *minTermSize* (p=1.0 $\times 10^{-11}$). The SO is the one of two ontologies where there is a difference between the values of MM's *model* parameter. Using the *relaxed* model in place of *strict*, decreases P 0.1-0.3 with no change in R. We find that ~400 more FP are introduced for the best performing parameter combination when *relaxed* is used. A majority of the errors are from matching a capital letter at the beginning or end of a token. For example, "HKI" is incorrectly annotated with "SO:0001230 - inosine" and "SO:0001438 - isoleucince", both of which have "I" as a synonym. An error seen that was not due to matching capital letters is "DNA-binding" and "DNA binding" incorrectly annotated with "SO:0000417 - polypeptide_domain", which has a synonym "DNA _bind". We can conclude that it is better to use the *strict* model with SO.

There is no difference between using *default* and *unique* values of the *acronymAbb* parameter, but there is a difference when using *all*. Comparing *default* and *all*, P is decreased 0-0.2 with a slight increase in R. ~20 more TPs and ~4,000 more FPs are found when using *all* acronyms and abbreviations. One example of a term correctly recognized is due to synonymous adjectives, "short interfering RNA" is correctly annotated with "SO:0000646 - siRNA", which has a synonym of "small interfering RNA". Some abbreviations have more than one meaning and are not found unless *all* is used, e.g. "PCR product(s)" is correctly annotated with 'SO:0000006 - PCR_product". Unfortunately, there are many terms that have possible ambiguous abbreviations. For instance, "protein(s)" is incorrectly annotated with "SO:0001439 - proline", which has the synonyms "P" and "Pro"; Also, "states" is incorrectly annotated with "SO:0000331 - STS".

Filtering terms based on length has the same results as with the NCBO Annotator parameter above; filtering out terms less than 5 characters decreases R by 0.1-0.2, so it is best to filter terms less than 1 or 3. Along the same lines, it is best to use all or most of the annotations returned by MM, so setting *scoreFilter* equal to 0 or 600 is suggested.

Concept Mapper parameters - SO

Four parameters were found to be significant: *searchStrategy* (p=7.4 $\times 10^{-8}$), *stemmer* (p=2.2 $\times 10^{-16}$), *stopWords* (p=1.7 $\times 10^{-4}$), and *synonyms* (p=2.2 $\times 10^{-16}$). As seen in many of the other ontologies before, stemming is useful for improving recall. With the SO, there is no difference between Porter or BioLemmatizer, but there is a difference between a stemmer and *none*. When using Porter over *none*, ~3,800 more TPs

are found along with ~5,300 more FPs. Along with variants, such as "genomic" and "genomically" correctly annotated with "SO:0001026 - genome", using a stemmer allows plurals to be found. Not all variants carry the same meaning as the original term. For instance, "SO:0000141 - terminator" refers to the sequence of DNA at the end of a transcript that causes RNA polymerase to fall off, while "terminal", "terminally", and "termination" all carry different meanings. Even though using a stemmer introduces more incorrect annotations than correct ones, F-measure is increased by 0.1-0.2.

Removing PubMed stop words has varying effects. For one group, an increase in P of 0.05-0.2 with no change in R is seen, but for the other one, slight decreases in P and R are seen. The maximum F-measure parameter combination falls in the latter group, for which ~25 less TPs and ~200 more FPs are found when using PubMed stop words. The correct annotations found when not using stop words and missed by removing stop words are masked by longer FPs. For instance, not removing stop words, "SO:0000151 - clone" and "SO:0000756 - cDNA" are both correctly annotated in the span "clone in these cDNA", but when removing PubMed stop words the entire span is incorrectly annotated with "SO:0000792 - cloned_cDNA" because "in" and "these" are not considered. Errors introduced are from the 9.3% of terms that contain stop words that are integral to their meaning. For example, "motif" is incorrectly annotated with "SO:0001010 - i_motif". For the best performance, it is best to not remove stop words.

Creating dictionaries with *all* instead of only *exact synonyms* allows ~400 more TPs to be found while introducing ~5,000 more FPs, which leads to a decrease in P of 0.1-0.4 with an increase in R of 0-0.05. Only two examples make up all of the correct annotations found: "domain(s)" correctly annotated with "SO:0000417 - polypeptide_domain" which has broad synonym "domain" and "signal(s)" correctly annotated with both "SO:0000725 - transit_peptide" and "SO:0000418 - signal_peptide", which both have broad synonym "signal"; both of these correct annotations are matches to broad synonyms. Of the errors introduced, over half, ~2,600, of the incorrect annotations are broad synonyms from the following two examples: "region(s)", "site(s)", "position(s)", and "positional" are incorrectly annotated with "SO:0000839 - polypeptide_region" (has broad synonyms "region", "positional", and "site") and "signal(s)" incorrectly annotated with "SO:0000725 - transit_peptide" and "SO:0000418 - signal_peptide" (has broad synonym "signal"). It is interesting that the same broad synonym, "signal", produces a ~30 TPs but many more FPs (~1,300). We can conclude that the correct annotations found do not outweigh the errors introduced, so it is best to create dictionaries with only *exact synonyms*.

Protein Ontology

NCBO Annotator parameters - PR

Only *wholeWordsOnly* (p=2.3 $\times 10^{-11}$) was found to be significant. Matching non-whole words introduces ~1,500 more TPs and ~270,000 more FPs. The TPs that were found contained some kind of punctuation. For example, "BRCA2" from the spans " BRCA2-independent", "RAD51-BRCA2", and "BRCA2^{+}" are correctly annotated with "PR:000004804 - breast cancer type 2 susceptibility protein". Many of the FPs found are from matching smaller synonyms within longer words. An example,"PR:000008207 - synaptic glycoprotein SC2", which has an exact synonym "TER", is incorrectly found ~7,000 times in words such as "determine", "promoter", and "anterior". It is best to not allow NCBO Annotator to match non-whole words.

MetaMap parameters - PR

Four parameters were found to be significant: *gaps* (p=2.2 $\times 10^{-16}$), *acronymAbb* (p=2.2 $\times 10^{-16}$), *scoreFilter* (p=2.2 $\times 10^{-16}$), and *minTermSize* (p=7.1 $\times 10^{-14}$). Inserting gaps when matching decreases P by 0.2 and increases R slightly; varying *gaps* on maximum F-measure parameter combinations finds ~100 more TPs while introducing ~12,000 more FPs. *gaps* provide MM the ability to skip tokens to find matches. For example, "cyclin 5" is correctly annotated with "PR:000005258 - cell division protein kinase 5", which has a synonym "cyclin-dependent kinase 5"; the four tokens, "-dependent kinase", are skipped to allow this match. Even though skipping tokens find some TPs, many more errors are found. Some errors are close matches, but less specific terms, such as "alpha-crystallin" incorrectly annotated with "PR:000005908 - alpha-crystallin B chain". Others found are completely wrong; for instance, the span "protein-1" can be annotated with any term as long as it contains "protein", "-", and "1", in that order. "PR:000010001 - protein lyl-1" and "PR:000009230 - multisynthetase complex auxiliary component p38", which has the synonym "protein JTV-1" are examples of terms incorrectly matched with "protein-1". Not using gaps produces the highest F-measure.

The maximum F-measure is obtained by using *scoreFilter* =600 and *minTermSize* =3 or 5. A majority of the terms matched in PR are at least 3 characters long. By filtering some correct annotations will be lost, such as "Rb" annotated with "PR:000013773: retinoblastoma-associated protein", but for the most part it is safe to filter out terms less than 3 characters.

Concept Mapper parameters - PR

Four parameters were found to be significant: *caseMatch* (p=6.1 $\times 10^{-6}$), *stemmer* (p=2.2 $\times 10^{-16}$), *findAllMatches* (p=1.1 $\times 10^{-9}$), and *synonyms* (p=5.3 $\times 10^{-16}$). PR is the only ontology where *caseMatch* is significant. The *caseMatch* value CASE FOLD DIGITS produces the highest F-measure. Only text that contains digits is folded to lower case. ~2,000 fewer TPs and ~40,000 fewer FPs are found when comparing folding only digits to folding everything. Some annotations are missed, for example, "MYOC", which is a synonym of "PR:000010873 - myocilin" is not matched with "Myoc". Errors introduced by folding everything are mainly from folding synonyms that are common english terms. For example, "TO" is a synonym of "PR:000016214 - tryptophan 2,3-dioxygenase". Just from looking at the synonym, it is hard to determine when and when not to fold cases. For maximum F-measure, it is best to only fold those with digits.

Not using any type of stemmer produces the highest precision and F-measure. Using BioLemmatizer increases R by finding ~2,000 more TPs but decreases P by finding ~100,000 more FPs. Using a stemmer allows plurals to be found. For example, "neurotrophins" is correctly annotated with "PR:000021998 - neurotrophin". Also, using a stemmer folds all text to lower cases; for example, "Shh" is correctly annotated with "PR:000014841 - sonic hedgehog protein", which has a synonym "SHH". Generating morphological and derivational variants also introduces many other errors. For instance, "PR:000008323 - general transcription factor II-I repeat domain-containing protein 1" has a synonym "BEN", that when put through BioLemmatizer gets turned into "been", "is", "are", "be", "being", "were" that are found incorrectly ~15,000 times in CRAFT. Folding everything also produces incorrect annotations, such as "SAC" getting incorrectly annotated with "PR:000003752 - adenylate cyclist type 10", which has a synonym "sAC". Using a stemmer finds many TPs, but the many FPs introduced outweigh the TPs.

Using ***all*** *synonyms* produces the highest F-measure; ~5,000 fewer TPs are found by only using *exact synonyms*. Because ***all*** is the best performance it tells us that the synonym list for PR is well maintained and does not contain many spurious synonyms. In addition, many of the related synonyms are synonyms for the corresponding genes of proteins which are equivalently annotated in CRAFT. An example of an annotation missed by using only *exact synonyms* is "Car2" correctly annotated with "PR:000004918 - carbonic anhydrase 2"; it has an exact synonym of "Ca2" and a related synonym of "Car2". It is best to use ***all*** *synonyms* for PR.

NCBI Taxonomy

NCBO Annotator parameters - NCBITaxon

Two parameters were found to be significant: *wholeWordsOnly* (p=2.2 $\times 10^{-16}$) and *minTermSize* (p=7.2 $\times 10^{-5}$). Matching non-whole words decreases P by 0.1 with a slight increase in R. Varying *wholeWordsOnly* on the maximum F-measure parameter combination finds ~15 more TPs and ~5,500 more FPs. All correct annotations found contain connected punctuation that hinder recognition. For example, "Danio rerio" from the span "(Danio rerio [Dr])" is correctly annotated with "NCBITaxon:7955 - Danio rerio". Unfortunately, many errors are introduced by matching terms within longer words. For instance, the genus of bony fish, "NCBITaxon:385272 - Conta", is seen within "contain" and its variants. It is suggested to only allow matching to whole words.

Filtering terms that are less than *five* characters leads to the best performance on NCBITaxon, increasing P by 0.1 with no loss of recall over other parameter values. Comparing lengths of *one* to *five*, ~250 more FPs are found when not removing terms less than *five* characters. For example, "lens" is incorrectly annotated with the genus of flowering plants "NCBITaxon:3863 - Lens". For the reasons stated in the previous paragraph on recall of NCBITaxon, NCBO Annotator does not find any correct annotations that are less than *five* characters.

MetaMap parameters - NCBITaxon

Four parameters were found to be significant: wordOrder (p=1.4 $\times 10^{-6}$), *derivationalVariants* (p=1.0 $\times 10^{-8}$), *scoreFilter* (p=2.2 $\times 10^{-16}$), and *minTermSize* (p=2.2 $\times 10^{-16}$). Allowing MM to ignore the order of tokens varies P slightly but decreases R by 0-0.3. Unfortunately, changing wordOrder on the maximum F-measure combination only introduces ~5 more FPs, so the full effect of the parameter is not really seen. Even though the effect cannot be seen, keeping the order of the tokens produces the maximum F-measure.

Generating variants of terms helps performance of most other ontologies evaluated, but not using any derivational variants produces highest F-measure for NCBITaxon. Allowing MM to use variants decreases P 0.05-0.1 with only slight increase in R. Using *adj noun only* variants finds ~150 more TPs along with ~5,000 more FPs. There are some cases where variants are valid, such as "mammalian" correctly annotated with "NCBITaxon:40674 - Mammalia". For the the most part, nomenclature variants do not follow the same rules for English words. For example, a genus name of birds is "NCBITaxon:189528 - Indicator"; when variants of this are generated the words "indicate(s)", "indicated", "indicating", and "indication" are incorrectly annotated with it. Even though *derivationalVariants* are not used, variants such as "mice"

→ "mouse" are still correctly found; this shows that inflectional variants are apparently handled by MM even when *derivationalVariants* are not used and suggests that this behavior cannot be controlled with a parameter. For best performance on NCBITaxon, do not use any variants.

Unlike NCBO Annotator, it is best to filter terms less than 1 or 3 characters in length. There is no difference between removing terms less than 1 or 3, but filtering terms less than 5 decreases R by 0.1-0.6. This is the case because many correct annotations found, e.g. "mice", are less than 5 characters.

Concept Mapper parameters - NCBITaxon

All but one parameter were found to be significant: *searchStrategy* (p=3.9 $\times 10^{-9}$), *caseMatch* (p=9.9 $\times 10^{-14}$), *stemmer* (p=2.2 $\times 10^{-16}$), *stopWords* (p=3.5 $\times 10^{-4}$), *findAllMatches* (p=2.2 $\times 10^{-16}$), and *synonyms* (p=2.9 $\times 10^{-7}$). *caseMatch* is an interesting parameter; for the best performing combinations, it does not matter because BioLemmatizer is used and it natively changes everything to lower case. Also, allowing CM to find all matches, instead of only the longest one, leads to a decrease in P of 0.3-0.4 with only a slight increase in R.

The maximum F-measure by CM uses BioLemmatizer as a *stemmer*. An increase in R of 0.2 and varying effects on P are seen by using BioLemmatizer over *none* or Porter. ~1,700 more TPs and ~2,000 more FPs are found by varying BioLemmatizer vs *none* on the maximum F-measure combination. A majority of the correct annotations found (~1,100) are from the variant "mouse" being correctly normalized to "NCBITaxon:10088 - Mus". Not all variants generated are correct. For example, "area" is incorrectly annotated with "NCBITaxon:293506 - Areae" and the gene that controls coat color, "agouti", is an incorrect variant of "agoutis", which is the common name of "NCBITaxon:34845 - Dasyprocta". Even though more FPs are found, the increase in R outweighs the loss of P and a total increase in F of 0.07 is seen.

Removing PubMed stop words produces differing results; for some parameter combinations there is an increase in P of 0.05-0.2 and for others there is a decrease in P of 0-0.05, while stop words doesn't seem to effect R. Not removing stop words finds ~2,600 more incorrect annotations. A majority (~1,800) of the errors introduced by not removing stop words are due to the word "this" being incorrectly annotated with "NCBITaxon:169495 - This". Not removing stop words and then allowing stemming introduces errors as well. For example, "can", "could", and "cannot" are incorrectly annotated with "NCBITaxon:4627 - Canna". Removing stop words produces the highest F-measure because these common English words are ignored.

ChEBI

NCBO Annotator parameters - ChEBI

Only *wholeWordsOnly* (p=2.3 $\times 10^{-11}$) was found to be significant. Like all other ontologies above, it is best to only match whole words. When allowing to match non-whole words, P decreases 0.4-0.6 with a slight decrease in R. ~500 more TPs and ~36,000 more FPs are found when NCBO Annotator recognizes non-whole words. Correct annotations found by matching non-whole words contain punctuation. For example, "CHEBI:37527 - acid" and "CHEBI:30879 - alcohol" are correctly found in "Fekete's acid-alcohol-formalin fixative". ChEBI contains small terms that are found within longer words such as "CHEBI:24870 - ion", which is found incorrectly ~17,000 times in words such as "proliferation", "mutation", and "localization". Also many errors are introduced from mixing synonyms and matching non-whole words. For instance, "CHEBI:27007 - tin atom" has a synonym "tin", which is found ~4,000 times within words like "blotting", "continious", and "intestinal". Both of these examples are small and would be filtered out if *minTermSize =five* was used, but there are also examples that are longer; for example, "CHEBI:35701 - ester" is incorrectly found within "cholesterol" and "western". Overall, it is best to not match non-whole words.

MetaMap parameters - ChEBI

Four parameters were found to be significant: *model* (p=2.4 $\times 10^{-10}$), *acronymAbb* (p=2.2 $\times 10^{-16}$), *scoreFilter* (p=2.2 $\times 10^{-16}$), *minTermSize* (p=7.2 $\times 10^{-14}$). ChEBI is one of two ontologies where a difference is seen between values of the *model* parameter. Using the *strict* model instead of *relaxed* increases P 0-0.5 with no change in R, which leads to an increase in F-measure of 0-0.1. Changing the best parameter combination's model to *relaxed* finds ~200 more FPs with no more TPs. It is unclear why the errors seen are thought to be correct by MM. For example, the text "Ndrg1", which looks to be a protein name, is incorrectly annotated with terms like "CHEBI:30226 - azanldylidene group", "CHEBI:33268 - monoatomic nitrogen", and "CHEBI:36934 - nitrogen-15 atom". The only thing in common between those three ChEBI terms is they all have a synonym of "N". To achieve the best performance, the *strict* model should be used.

For best performance, terms less than 5 characters should be filtered out. By doing this P is increased 0.3-0.5, but R is decreased by 0.2; F-measure is increased by 0.05. Comparing the lengths of terms filtered (3 vs. 5), we find that ~1,000 TPs are missed but ~8,000 FPs are avoided. It makes sense that the TPs missed are terms and abbreviations that are 3-4 characters in length such as "CHEBI:37527 - acid", "CHEBI:33290 - food", and "EDTA", which is a synonym of "CHEBI:42191 - ethylenediaminetetraacetic acid". The errors filtered out are mostly due to synonyms that contain ambiguous abbreviations. For example

“PGC” is incorrectly annotated with “CHEBI:26336 - prostaglandins C” and “male” is incorrectly annotated with “CHEBI:30780 - maleate(2-)”. Along the same lines, the *acronymAbb* parameter can introduce many more erroneous abbreviations if the value is set to ***all***. In order to minimize errors introduced through abbreviations, it is best to use set *acronymAbb* to ***default*** or *unique* and to also set *minTermSize* to filter out 5 or less characters.

Concept Mapper parameters - ChEBI

Only one parameter was found to be statistically significant, *synonyms* ($p=2.2 \times 10^{-16}$). This does not mean that this parameter is the only one that matters and that any combination will perform well. What we see happening is that the *synonyms* parameter separates the data into two distinct groups and that the effect of other parameters on each group is widely different. For example, we find that *stemmer* performance is directly tied to which synonyms are used. When ***all*** *synonyms* are used, there is no difference between any of them, but when using ***exact*** *synonyms*, the stemmers cluster into three distinct groups, with BioLemmatizer achieving the best performance.

Using ***all*** *synonyms* decreases P by 0.4-0.6 with varying effects on R. Examining the highest F-measure performing combination, ~1,000 more TPs and ~365,000 more FPs are introduced by creating the dictionary with ***all*** *synonyms* instead of ***exact***. Correct annotations found are mostly from abbreviations. For example “NaCl” is correctly annotated with “CHEBI:26710 - sodium chloride”, “MgSO4” is correctly annotated with “CHEBI:32599 - magnesium sulfate”, and “mRNA” is correctly annotated with “CHEBI:33699 - messenger RNA”. Abbreviations for chemicals can introduce many errors; for example, “CHEBI:30430 - indium atom” and “CHEBI:30433 - indium(1+)” both have a synonym of “In”, which is a common English word and seen ~56,000 times in CRAFT. Mixing ***all*** *synonyms* and any stemmer produces interesting errors also. For example, “CHEBI:33783 - beryllium(0)” has a synonym “Be”, which is incorrectly annotated to “am”, “is”, “are”, “was”, “been”, etc... We can conclude that the non-exact synonyms for ChEBI are not helpful for concept recognition.
