# Supplementary material for: Large-scale biomedical concept recognition: an evaluation of current automatic annotators and their parameters
Source: BMC Bioinformatics. 2014 Feb 26;15:59. doi: 10.1186/1471-2105-15-59 (PMC4015610; doi:10.1186/1471-2105-15-59)
Supplement: Additional file 3 — Comparison of CM to ChemSpot. Comparison between ConceptMapper and ChemSpot, a ChEBI specific named entity recognition tool. It was performed and written up as a lab rotation by Benjamin Garcia. [file 1471-2105-15-59-S3.pdf]

Analysis of the ChemSpot tool for recognizing ChEBI terms in CRAFT

Introduction

ChemSpot is a chemical named entity recognition (NER) tool with both a machine learning and a dictionary component [1B]. As a specialized NER tool, it is only applicable to recognizing the terms in the ChEBI ontology and therefore not appropriate to the primary study of the main paper, which focuses on the performance of general-purpose tools for term recognition. Here, we investigate the performance of ChemSpot on the ChEBI terms annotated in CRAFT, and contrast ChemSpot with the performance of the ConceptMapper (CM) tool.

The ChemSpot machine-learning component utilizes BANNER [2B] to learn IUPAC names, and the dictionary component utilizes ChemIDplus [3B] with LINNEAUS [4B], as shown in Figure AF1. The machine-learning component is preferentially chosen over the dictionary due to more accurate boundary detection [1B]. When generating entities from the dictionary, the union of overlapping matches is taken [1B]. This union is not ideal for ChEBI matching, an occurrence of “amino acid” in CRAFT is annotated with four different ChEBI terms (“CHEBI:46882 - amino group”, “CHEBI:32952 - amine”, “CHEBI:37527 - acid”, and “CHEBI:33708 - amino-acid residue”.). Neither the dictionary nor the IUPAC model were altered for these experiments, in order to preserve the intended functionality of ChemSpot.

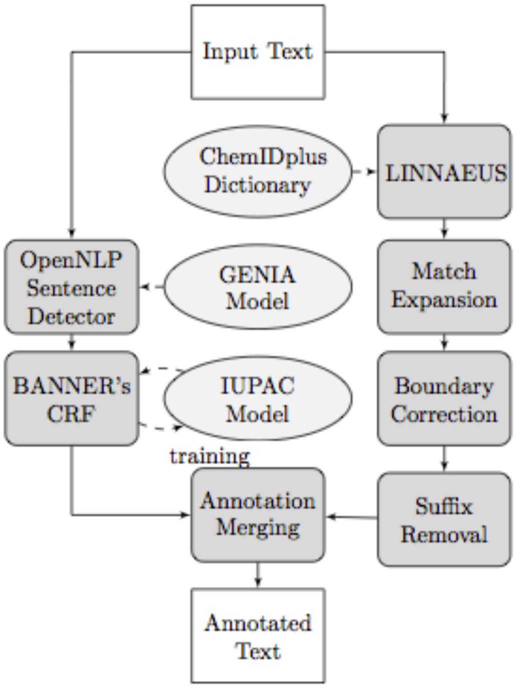

Figure AF1. ChemSpot pipeline, where input text is read in, evaluated by both the dictionary and machine-learning tools, and then applied for annotation of chemical entities.

Methods

ChemSpot was applied to find chemical entities within CRAFT. Entities found by ChemSpot are evaluated against the CRAFT gold standard for precision, recall, and F-measure. The ChemSpot v0.906 source and the ChemSpot dictionary file utilized for this analysis were downloaded on May 10th, 2012 [1B]. A UIMA wrapper was implemented for ease of use within the existing concept recognition evaluation pipeline.

ChemSpot annotations could not be evaluated directly against ChEBI IDs in the CRAFT annotations, as ChemSpot does not consistently provide specific identifiers. Therefore, only the presence or absence of an annotation at a particular document span was evaluated. CM has stricter matching criteria than ChemSpot because it must match the text span and ChEBI ID, whereas ChemSpot must only match the span. Both CM and ChemSpot output was evaluated using both strict and overlap spans, with overlapping spans having a higher F-measure. All numbers presented here are from the overlap comparison.

The parameters that were provided to CM for this evaluation were: SearchStrategy: SKIP_ANY_MATCH_ALLOW_OVERLAP; CaseMatch: CASE_SENSITIVE; Stemmer: NONE; Stopwords: PUBMED; OrderIndependentLookup: OFF; FindAllMatches: NO.

Results

Overall

For chemical entities, CM had a 0.046 higher F-measure. CM had over twice as many true positives, but also over three times as many false negatives. This is reflected in higher recall for CM but lower precision, as seen in Table AT1. An error analysis was performed on the 20008 false positives and false negatives for CM, and the 10954 false positives and false negatives for ChemSpot. This difference of 1040 between CM and ChemSpot can be attributed to the fact that CM allows for substring matching but ChemSpot does not.

	CM	ChemSpot
True Positives	5528	2461
False Positives	16133	5052
False Negatives	3875	5902
Precision	0.255	0.328
Recall	0.588	0.294
F-measure	0.356	0.31

Table AT1. True positives, false positives, and false negatives for CM and Chemspot, as well as their corresponding precision, recall, and F-measure.

Four categories were used for error analysis: *Entities*, *Boundaries*, *String Variation*, and *Generics*. *Entities* refers to names of specific chemicals, genes, and people/places. *Boundaries* refers to errors in span matching. *String Variation* refers

to short strings, and non-alpha-numeric characters. *Generics* are strings that either describe entities or are general categories for entities.

False Positives

For ConceptMapper, false positives could indicate either a mismatch of ChEBI IDs between the annotation and gold standard or a lack of corresponding span in the gold standard. For ChemSpot, all false positives indicate only a lack of overlap between a ChemSpot annotation and a gold standard annotation. The number of annotations in each category can be seen in Figure AF2, where Total Unique represents the number of unique spans.

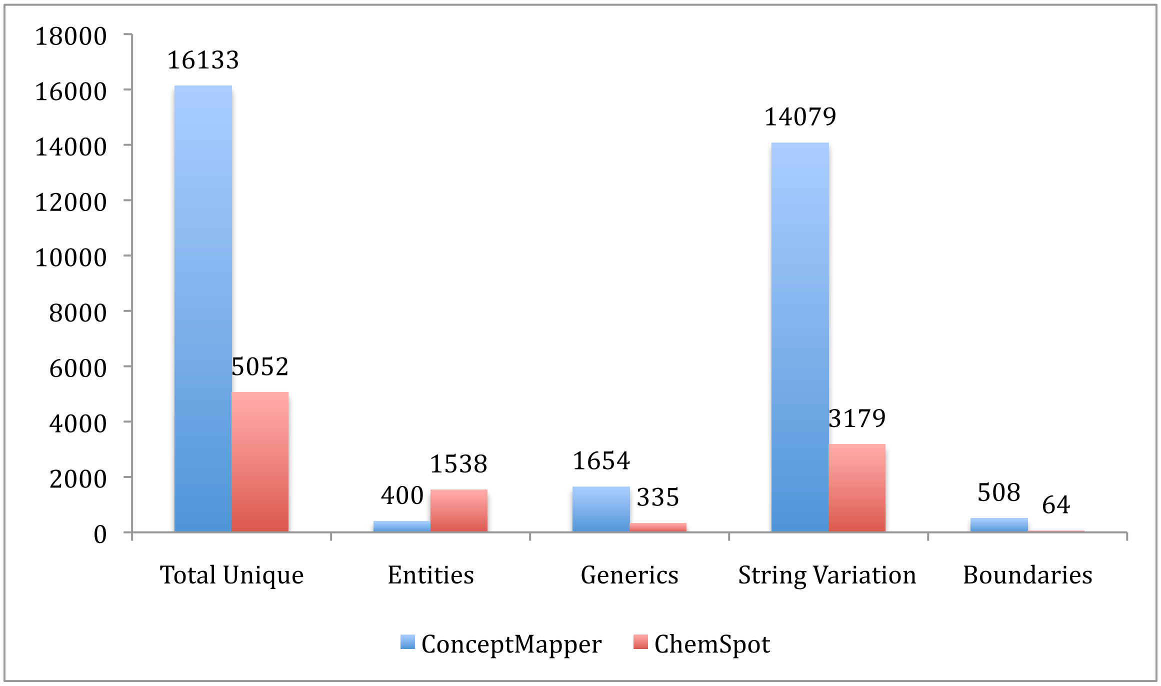

Figure AF2. Total number of false positive annotations and their categories for chemical annotations produced by CM and ChemSpot.

CM

Most of the false positives (87%) for CM are *String Variation*, with half of these being single numbers. These single numbers were given ChEBI annotations representing chemicals with charges. In one case, "0" represented beryllium (0), and in another case, "3" represented any 3+ atom. Adding single numbers to the stopword list would increase precision from 0.255 to 0.381 and F-measure from 0.356 to 0.462. It is inappropriate to add single letters to the stopword list as there were single letters, representing chemicals, in the CRAFT gold standard. The conjunction "or" was annotated 1826 times with the ChEBI ID for gold, as it is the French word for gold. This word is not in the PubMed stopword list that was used for this analysis. Adding the conjunction to the stopword list would increase precision from 0.255 to 0.279 and recall from 0.356 to 0.378. A list of specific categories making up greater than 1% of the total false positives for CM can be seen in Table AT2.

CM produced two other false positives of note. There were 698 strict span matches between false positives and false negatives. These differences represent differences in ChEBI IDs produced by CM. One example: “in stria vascularis. Cys, **cysteine**, Glu, glutamate, Gly, glycine,” the cysteine shown in bold was annotated by CM with cysteinyl radical. However, the correct annotation, based on the gold standard, should have been cysteine. The second false positives of note were 138 sentence fragments being annotated as chemical. In the example: “B) Amplification products of cDNA from the following tissues”, was annotated with Boron (3+).

Category	Number	Percent	Examples
number	7136	44.2%	"0", "1", "2"
letter	2181	13.5%	"N", "O", "B and C"
conjunction	1826	11.3%	"or"
abbr/acronym	1730	10.7%	"Ala", "CO2", "TNT"
descriptor	1348	8.4%	"buffer", "group", "male"
number/letter	408	2.5%	"5B", "H2O2", "S8"
part of chemical	363	2.3%	"chloro", "methyl", "N-ethyl-N"
chemical	208	1.3%	"Acylcarnitine", "oxygen", "rotenone"
other	933	5.8%	amino acids, sentence fragments, measurements, etc
**total**	**16133**	100%	

Table AT2. CM false positive categories. “Other” is a collection of categories that make up less than 1% of the total number of false positives.

Category	Number	Percent	Example
abbr/acronym	2419	47.9%	"AAA", "CIA", "mon"
chemical	647	12.8%	"7-dehydrocholesterol", "Alcian blue", "forskolin"
gene	636	12.6%	"BrdU", "integrin", "placental lactogen-1"
descriptor	297	5.9%	"apex", "citation", "smear"
dash	278	5.5%	"D-2", "seizure-prone", "counter-stained"
number/letter	244	4.8%	"2 h", "at 7", "P21"
name	128	2.5%	"Aurora", "Franklin", "Pediatrix"
strain	103	2.0%	"CBA/CaHN-Btkxid/J", "tet-APPswe/ind", "XpdTTD/TTD"
address	75	1.5%	"http://genome.ucsc.edu", "doi:10.1371/journal.pgen.0020216.eor"
part of chemical	56	1.1%	"fluor", "parrafin"
other	172	3.4%	chemical descriptors, measurements, 5'region, etc
**total**	**5052**	100%	

Table AT3. ChemSpot false positive categories. “Other” is a collection of categories that make up less than 1% of the total number of false positives.

ChemSpot

Slightly less than half (48%) of the false positives produced by ChemSpot were based on *abbreviations and acronyms*. These are strings that are difficult to annotate, as their context is highly dependent upon the strings around the annotation. ChemSpot also had difficulty with over-annotating *genes/proteins* and *strings containing dashes* as chemicals. Dashes are common in IUPAC names, the machine learned aspect of ChemSpot. As such, the machine learning part of ChemSpot annotated many dashed strings as if they were IUPAC names. The list of categories making up greater than 1% of the total false positives for ChemSpot can be seen in Table AT3.

There are 703 chemicals and parts of chemicals with no overlapping span match between ChemSpot annotations and CRAFT gold standard annotations. These ChemSpot annotations represent differing annotation guidelines in CRAFT or elements that lack ChEBI IDs. In the example: "Upon stimulation with **forskolin**, a cAMP-dependent protein kinase activator, wild-type AQP2 translocated to the apical surface of polarized MDCK cells (Figure 4B, second column).", the forskolin shown in bold was annotated by ChemSpot but lacks an annotation in CRAFT. The ChEBI ID for this chemical is CHEBI:42471, indicating differing annotation guidelines in the CRAFT gold standard or a different version of ChEBI. If all of these chemicals are believed to be true the maximum possible precision would increase from 0.328 to 0.421, recall would increase from 0.249 to 0.349, and F-measure would increase from 0.31 to 0.381.

Comparison

The majority of errors in both CM and ChemSpot were String Variation. For CM, half of these false positives can be easily dealt with by additions to the stopword list. However, for ChemSpot there is no simple way to reduce these false positives. False positive *chemicals* and *parts of chemicals* found by CM represent mostly errors attributed to the CM methods. On the other hand, the false positives found by ChemSpot, especially the ones with ChEBI IDs, represent possible errors in the CRAFT gold standard annotations or different version of ChEBI used.

False Negatives

False negatives represent the number of gold standard annotations that did not have a corresponding named entity recognizer annotation. For CM, these annotations could be mismatches of ChEBI identifiers between the tool annotations and CRAFT, or could represent strings that did not map to dictionary entries. For ChemSpot, most of the false negatives correspond to *generics*, many of which ChemSpot was not designed to find. The number of annotations in each category can be seen in Figure AF3, where Total Unique represents the number of unique spans.

CM

False negatives in CM were evenly distributed across many categories, with *chemicals*, *parts of chemicals*, and *other chemical entities* making up 30% of the total false negatives. *Descriptors* and *chemical descriptors* made up 27% for false negatives, *short strings* made up 25% of false negatives, with *generic chemical*

references making up most of the remaining 18% of false negatives. The list of categories with number of elements making up greater than 1% of the total false positives for CM can be seen in Table AT4.

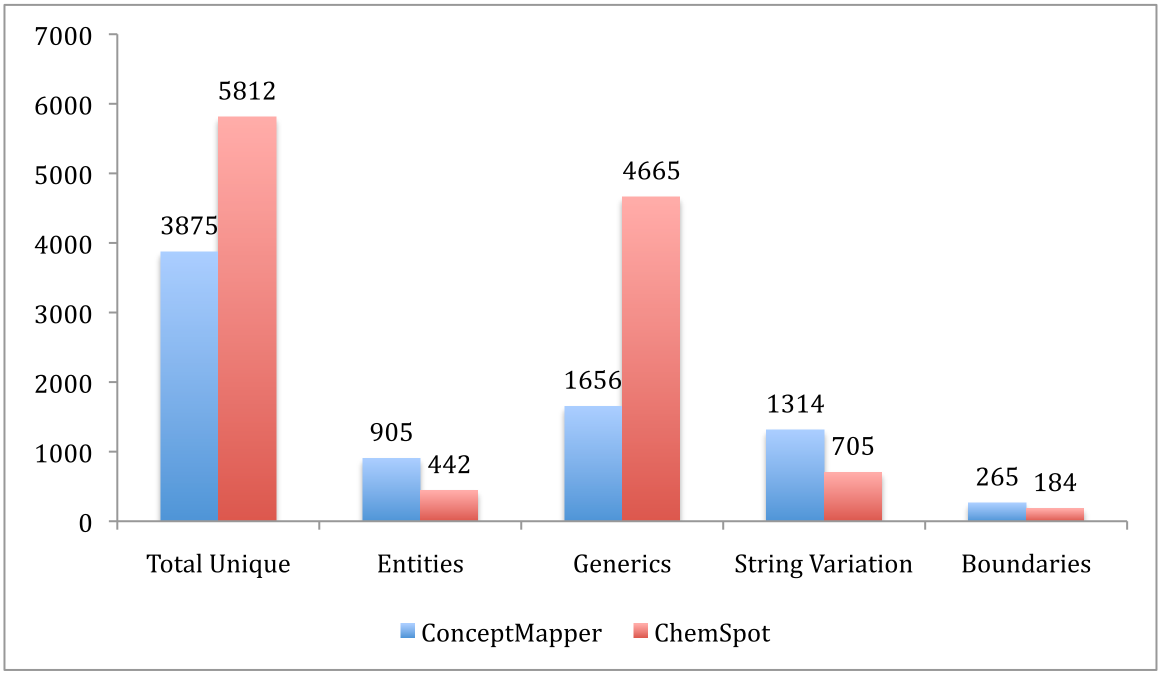

Figure AF3. Total number of false negative annotations and their categories for chemical annotations produced by CM and ChemSpot.

Category	Number	Percent	Example
descriptor	562	14.5%	"chain", "inhibitor", "tagged"
generic amino acid	518	13.4%	"amino acid", "amino", "amino acids"
chemical	490	12.6%	"cacodylate", "dithiothreitol", "n-hexane"
chemical descriptor	471	12.2%	"alkaline", "molecular", "surfactant"
letter	469	12.1%	"A", "C", "O"
abbr/acronym	464	12.0%	"AA", "EDTA", "UTP"
part of chemical	258	6.7%	"acyl", "citrate", "sulfate"
ion	186	4.8%	"Cl-", "K+", "NAD+"
amino acid	142	3.7%	"cysteine", "D-lysine", "glutamate"
generic nucleic acid	105	2.7%	"nucleotide", "cyclic nucleotide", "oligonucleotide"
DNA/RNA	96	2.5%	"DNA", "ssDNA", "mRNA"
other	114	2.9%	nucleic acids, chemical formulas, genes, etc
**total**	**3875**	100%	

Table AT4. CM false negative categories. "Other" is a collection of categories that make up less than 1% of the total number of false positives.

ChemSpot

An overwhelming majority (80%) of ChemSpot false negatives are *generics*. Only 9% of false negatives were *chemicals*, *parts of chemicals*, and *other chemical entities*. This is due to ChemSpot being specifically designed for finding chemicals. The list of categories with number of elements making up greater than 1% of the total false positives for ChemSpot can be seen in Table AT5.

Category	Number	Percent	Example
protein(s)	1515	26.1%	"protein", "proteins", "peptide"
DNA/RNA	1119	19.3%	"dsDNA", "duplex DNA", "ribosomal RNA"
descriptor	834	14.3%	"agonist", "dye", "marker"
chemical descriptor	718	12.4%	"acidic", "drug", "surfactant"
generic amino acid	365	6.3%	"amino", "amino acid", amino acids"
letter	325	5.6%	"A", "N", "T"
chemical	274	4.7%	"cyclohexamine", "ethidium bromide", "thiamin pyrophosphate"
abbr/acronym	185	3.2%	"PS", "pro", "gly"
part of chemical	183	3.1%	"acyl", "acid", "silica"
generic nucleic acid	114	2.0%	"nucleic acid", "nucleotides", "oligonucleotide"
gene	91	1.6%	"cytochrome c", "haemoglobin", "myoglobin"
other	89	1.5%	ion, nucleic acid, etc
**total**	**5902**	100%	

Table AT5. ChemSpot false negative categories. "Other" is a collection of categories that make up less than 1% of the total number of false positives.

"Protein(s)" and "DNA"/"RNA" made up 45% of the false negatives; since ChemSpot was designed to find chemicals and not specifically ChEBI terms, it does not find these categories. The only strings found from these two categories contained a "-", an artifact of the machine learning tool for finding IUPAC names. Adding "protein", "proteins", "peptide", "DNA", and "RNA" to the ChemSpot dictionary has the potential to increase precision from 0.328 to 0.502, recall from 0.249 to 0.609, and F-measure from 0.31 to 0.551, thus greatly improving performance against the CRAFT gold standard.

Comparison

While ChemSpot has roughly 1.5x the number of false negatives as CM, ChemSpot has 2.5x fewer missed chemicals. The majority of ChemSpot false negatives are *generics*, while CM's false negatives are evenly distributed across all categories. This can be attributed to the fact that CM is designed to find ChEBI concepts; whereas, the focus for ChemSpot is on chemicals and small bio-molecules.

Conclusion

ChemSpot and CM cannot be strictly compared, due to differences in scope, but generalities can be identified. ChemSpot was specifically designed to find chemicals and is more accurate in finding *chemicals, parts of chemicals,* and *other chemical entities compared* to CM. In contrast, CM is a general concept recognition tool and is able to find more ChEBI terms when provided directly with the ChEBI dictionary.

Small changes to stopword lists and dictionaries can have a huge impact on F-measure. By adding single digit numbers and the term "or" to the CM stopword list, the F-measure increases by 40%. When adding "protein(s)", "peptide", "DNA" and "RNA" to the ChemSpot dictionary, the F-measure increases by 78%. The summation of these changes can be seen in Table AT6. Additionally, ChemSpot false positive categories *chemicals, parts of chemicals,* and *other chemical entities* can be utilized to assess the comprehensiveness of the CRAFT annotation gold standard. These false positives represent chemicals with no span match in the gold standard, meaning a potential missed annotation, lack of ChEBI term, or differeing versions of ChEBI (allowing for additions to the ChEBI ontology).

	CM	CM Changes	ChemSpot	CS Changes
Precision	0.255	0.435	0.328	0.502
Recall	0.588	0.587	0.294	0.609
F-measure	0.356	0.5	0.31	0.551

Table AT6. Precision, recall and F-measure for CM and ChemSpot. CM Changes represents inclusion of "0"-"9" and "or" to the CM stopword list. CS Changes represent inclusion of "protein", "proteins", "peptide", "DNA" and "RNA" to the dictionary.

References

[1B] Rocktäschel, T., Weidlich, M., Leser, U. (2012).ChemSpot: A hybrid system for chemical nemed entity recognition. Bioinformatics 10.1093

[2B] Leaman, R. and Gonzalez, G. (2008). BANNER: An executable survey of advances in biomedical named entity recognition. In Proceedings of the Pacific Symposium on Biocomputing, volume 13, pages 652-663.

[3B] Hettne, K., Stierum, R., Schuemie, M., Hendriksen, P., Schijvenaars, B., Mulligen, E., Kleinjans, J., and Kors, J. (2009). A dictionary to identify small molecules and drugs in free text. Bioinformatics, 25(22).

[4B] Gerner, M., Nenadic, G., and Bergman, C. (2010). LINNAEUS: A species name identification system for biomedical literature. BMC Bioinformatics, 11(1).
